# Racial Differences in ctDNA Profiles, Targeted Therapy Use, and Outcomes in Metastatic Breast Cancer

**DOI:** 10.1001/jamanetworkopen.2024.61899

**Published:** 2025-02-26

**Authors:** Emily L. Podany, Lorenzo Foffano, Lorenzo Gerratana, Arielle J. Medford, Katherine Clifton, Shaili Tapiavala, Marko Velimirovic, Marla Lipsyc-Sharf, Carolina Reduzzi, Adrian Bubie, Annika Putur, Foluso O. Ademuyiwa, Fabio Puglisi, William J. Gradishar, Cynthia X. Ma, Aditya Bardia, Massimo Cristofanilli, Andrew A. Davis

**Affiliations:** 1 Department of Medicine, Washington University in St Louis, St Louis, Missouri; 2 Department of Medicine, University of Udine, Udine, Italy; 3 Department of Medical Oncology, CRO Aviano, National Cancer Institute, IRCCS, Aviano, Italy; 4 Department of Medicine, Massachusetts General Hospital Cancer Center, Boston; 5 Department of Medicine, Cleveland Clinic, Cleveland, Ohio; 6 Department of Medicine, UCLA Health, Los Angeles, California; 7 Department of Medicine, Weill Cornell Medicine, New York, New York; 8 Guardant Health, Palo Alto, California; 9 Department of Medicine, Northwestern University, Chicago, Illinois

## Abstract

**Importance:**

Black patients with metastatic breast cancer (mBC) have higher mortality rates than White patients despite advances in treatment.

**Objectives:**

To examine whether Black patients with metastatic breast cancer have different genomic profiles compared with White patients and whether there are inequities in targeted treatment use between these groups.

**Design, Setting, and Participants:**

This retrospective, population-based cohort study assessed adult patients with mBC who underwent genomic profiling at academic institutions in the US between January 1, 2015, and December 31, 2023. Data analysis was performed between July 2023 and July 2024. A validation cohort was also included.

**Exposures:**

Targeted treatment use.

**Main Outcomes and Measures:**

The main outcomes were differences in circulating tumor DNA profiles and use of phosphoinositide 3-kinase (PI3K), mammalian target of rapamycin (mTOR), and cyclin-dependent kinase 4/6 (CDK4/6) inhibitors between Black and White patients with metastatic breast cancer.

**Results:**

The study sample included 1327 women with mBC (mean [SD] age, 58.0 [12.8] years; 140 Black and 1057 White). Black patients had a significantly higher rate of *GATA3* single-nucleotide variants (odds ratio, 2.31; 95% CI, 1.17-4.54; *P* = .02) and *CCND2* copy number variants (odds ratio, 4.63; 95% CI, 1.79-11.97; *P* = .002) on multivariate analysis. These differences were validated in a population-based evidence cohort of 27 224 patients. Black patients with *PIK3CA* single-nucleotide variants were significantly less likely to receive PI3K inhibitors than White patients (1 of 17 [5.9%] vs 45 of 156 [28.8%]; *P* = .04), whereas there was no difference in use of CDK4/6 and mTOR inhibitors, which do not require a targetable alteration. Black patients had a shorter overall survival from the time of circulating tumor DNA testing compared with White patients.

**Conclusions and Relevance:**

This cohort study of patients with mBC found somatic differences, shorter overall survival, and targeted treatment disparities in PI3K inhibitor use in Black compared with White patients despite equal incidence of *PIK3CA* alterations. Researchers should consider these differences when designing future research and interventions to address the striking and persistent outcomes gap between Black and White patients with mBC.

## Key Points

**Question:**

Are there differences in circulating tumor DNA (ctDNA) profiles and targeted therapy use between Black and White patients with metastatic breast cancer (mBC)?

**Findings:**

In this multi-institution cohort study of 1327 patients with mBC, there were significant differences in ctDNA profiles between Black and White patients. Black patients were significantly less likely to receive phosphoinositide 3-kinase inhibitors (5.9% vs 28.8%), despite equal incidence of *PIK3CA* alterations.

**Meaning:**

These findings suggest that clinical inequities exist alongside genomic differences; researchers must consider both when designing future research and interventions to address the outcomes gap between Black and White patients with mBC.

## Teaser

This cohort study evaluates whether genomic profiles and targeted treatment use differ between Black and White patients with metastatic breast cancer.

## Introduction

The stark disparities in breast cancer treatment outcomes and survival in Black compared with White patients in the US are well documented.^1,2,3,4,5,6^ Black patients have a 40% higher breast cancer–specific mortality when compared with White patients, present at a later stage,^7^ and are less likely to be represented in clinical trials.^8,9,10,11,12^ These disparities are multifactorial, with socioeconomic factors and clinicopathologic differences potentially contributing. Prior studies evaluating somatic genetic differences among patients with breast cancer have primarily focused on tumor tissue analysis. However, these studies may be limited by the known higher intratumor genetic heterogeneity of breast tumors from Black patients.^13,14^

Circulating tumor DNA (ctDNA) testing is a noninvasive, highly sensitive, and specific approach to detect heterogenous somatic alterations in metastatic breast cancer (mBC) and guide treatment based on mutational profiles and clonal evolution.^15,16,17,18,19,20,21,22,23,24,25^ There is relatively high concordance between ctDNA and tissue genetic findings in mBC, especially at higher allelic frequencies and ctDNA fractions.^15,26,27,28^ ctDNA has recently become incorporated into standard practice to assess somatic alterations; however, the evaluation of differences in ctDNA profiles between Black and White patients with mBC is limited. In this study, we evaluated differences in genomic profiles in a large, diverse, multi-institutional, clinically annotated database of patients with mBC and ctDNA profiles to assess racial differences in somatic profiles and clinical outcomes. We validated these differences using a large, population-based genomics database. We then evaluated targeted treatment use and clinical trial enrollment by race.

## Methods

### Patient Selection and Study Design

The institutional review boards at Washington University in St. Louis, Northwestern University, and Massachusetts General Hospital approved this study. The study was retrospective, and informed consent was therefore waived. The study was conducted in accordance with the Health Insurance Portability and Accountability Act. All patients with mBC who underwent ctDNA profiling using the commercially available Guardant360 assay (Guardant Health) from January 1, 2015, to December 31, 2023, were identified at each site. ctDNA results were examined for number and type of somatic gene alterations, variant allelic frequency (VAF), and date of collection. Clinical information gathered included metastatic disease sites, pathologic data, patient-reported race and ethnicity, prior treatments, and progression-free survival for each treatment line and overall survival (OS) from date of initial ctDNA collection. Ancestry data were not available. All clinical and genomic information were deidentified and combined into a Health Insurance Portability and Accountability Act–compliant database. Missing data were excluded. This retrospective cohort study adheres to the Strengthening the Reporting of Observational Studies in Epidemiology (STROBE) reporting guideline.

### ctDNA Testing and Real-World Validation Cohort

The Guardant360 assay detects single-nucleotide variants (SNVs), insertions, and deletions in up to 83 genes, copy number variants (CNVs) in 19 genes, and fusions in 11 genes. The analytical sensitivity and threshold for detection have been previously described.^29,30^ Guardant360 reports synonymous variants, pathogenic variants, and variants of uncertain significance. We defined oncogenic pathways based on prior work with The Cancer Genome Atlas^31^ and narrowed our pathway analysis to include only genes detectable with the Guardant360 assay.

GuardantINFORM is a clinicogenomics database that includes aggregated payer claims from outpatient and inpatient settings across community and academic practices and deidentified genomic data from Guardant Health assays. We used this database to create a validation cohort to assess somatic genomic differences by self-reported race in patients with mBC. Races were filtered to Black and/or African American (n = 3774) and White (n = 23 450). All alterations were included in the analysis if they were of somatic origin, regardless of pathogenicity.

### Targeted Treatment Analysis

We evaluated receipt of phosphoinositide 3-kinase (PI3K), mammalian target of rapamycin (mTOR), and cyclin-dependent kinase 4/6 (CDK4/6) inhibitors, either through clinical trial enrollment or after US Food and Drug Administration approval, by race. For PI3K inhibitors, we analyzed only patients with targetable *PIK3CA* (OMIM 171834) alterations. The SOLAR-1 trial (Study Assessing the Efficacy and Safety of Alpelisib Plus Fulvestrant in Men and Postmenopausal Women With Advanced Breast Cancer Which Progressed on or After Aromatase Inhibitor Treatment) required a glycated hemoglobin level (HbA_1c_) of 6.4% (to convert to proportion of hemoglobin, multiply by 0.01) or less as an inclusion criterion, so we assessed available HbA_1c_ data for the patients with *PIK3CA* alterations.^32^ We used the HbA_1c_ level closest in time to the baseline ctDNA testing with a cutoff of 3 months after baseline ctDNA evaluation.

### Statistical Analysis

Clinical and pathologic variables were reported using descriptive analyses through numbers and percentages for categorical variables or medians and IQRs for continuous variables. Clinical characteristics and genetic profiles were compared using the Fisher exact test to assess differences in alteration frequency across Black and White patients. Univariate and multivariate logistic regression for features associated with Black and White patients was performed to determine odds ratios (ORs) and 95% CIs. Gene alterations in both Black and White patients were analyzed through univariate models, and those with at least 5 pathogenic alterations were selected for multivariate regression when significant. For the population-based evidence validation cohort using GuardantINFORM, χ^2^ tests were used to determine differences across groups; multiple testing was corrected for using the Benjamini-Hochberg method. A 2-sided *P* < .05 was used to indicate significance.

We defined OS from the time of baseline ctDNA collection to death from any cause with data censored at last follow-up if the patient was still alive. Differences in OS were assessed using the log-rank test and Cox proportional hazards regression models and displayed using Kaplan-Meier plots. Only pathogenic alterations based on OncoKB were included in the logistic and Cox proportional hazards regression models. Statistical analysis was performed from July 2023 to July 2024 using Stata, version 16.1 (StataCorp LLC), JMP, version 16 (SAS Institute Inc), and R, version 4.1.0 (R Foundation for Statistical Computing).

## Results

### Patient Characteristics

A total of 1327 female patients (mean [SD] age, 58.0 [12.8] years) with mBC and ctDNA results between 2015 and 2023 were included in the cohort (474 from Washington University in St. Louis, 412 from Massachusetts General Hospital, and 441 from Northwestern University). Of the 1327 patients, 1196 (90.1%) self-reported Black or White race (135 of 1327 [10.2%] Black and 1061 of 1327 [80.0%] White). The remaining patients in the cohort self-identified as American Indian, Asian, or other race or did not report a race. We did not include these patients in the analysis due to the small number in each category. The Black and White patients’ tumors were categorized as hormone receptor positive (HR^+^)/ERBB2 (formerly HER2) negative (ERBB2^−^) (70.0%), ERBB2^+^ irrespective of HR status (13.9%), and triple negative (16.1%). A total of 243 patients (23.5%) had de novo mBC. There was no significant difference between Black and White patients in metastatic disease sites, presence of de novo mBC, number of prior treatment lines, endocrine therapy type, and subtype (Table 1 and Table 2).

**Table 1.** Characteristics of the Overall Cohort and by Race

Characteristic	No. (%) of patients^a^			Fisher test *P* value
	Overall (N = 1196)	Black (n = 135)	White (n = 1061)	
Estrogen receptor				
Positive	931 (78.1)	101 (74.8)	830 (78.5)	.33
Negative	261 (21.9)	34 (25.2)	227 (21.5)	
Progesterone receptor				
Positive	585 (49.8)	68 (51.1)	517 (49.7)	.75
Negative	589 (50.2)	65 (48.9)	524 (50.3)	
ERBB2				
Positive	156 (13.4)	16 (12.4)	140 (13.5)	.72
Negative	1007 (86.6)	113 (87.6)	894 (86.5)	
Histotype				
Ductal	803 (81.2)	95 (88.0)	708 (80.4)	.12
Lobular	136 (13.8)	11 (10.2)	125 (14.2)	
Mixed	50 (5.1)	2 (1.8)	48 (5.4)	
Liver metastases				
No	774 (64.8)	86 (63.7)	688 (65.0)	.77
Yes	420 (35.2)	49 (36.3)	371 (35.0)	
Lung metastases				
No	851 (71.3)	90 (66.7)	761 (71.9)	.21
Yes	343 (28.7)	45 (33.3)	298 (28.1)	
Central nervous system metastases				
No	1088 (91.7)	120 (88.9)	968 (92.1)	.20
Yes	98 (8.3)	15 (11.1)	83 (7.9)	
Bone metastases				
No	392 (32.8)	48 (35.6)	344 (32.5)	.47
Yes	802 (67.2)	87 (68.4)	715 (67.5)	
Nodal metastases				
No	751 (62.9)	80 (59.3)	671 (63.4)	.35
Yes	443 (37.1)	55 (40.7)	388 (36.6)	
Skin or soft tissue metastases				
No	981 (82.2)	110 (81.5)	871 (82.2)	.83
Yes	213 (17.8)	25 (18.5)	188 (17.8)	
Serosal metastases				
No	302 (88.6)	228 (88.4)	74 (89.2)	>.99
Yes	39 (11.4)	30 (11.6)	9 (10.8)	
De novo metastatic breast cancer				
No	790 (76.5)	80 (70.8)	710 (77.2)	.13
Yes	243 (23.5)	33 (29.2)	210 (22.8)	
Endocrine therapy type				
None	533 (50.1)	56 (47.9)	477 (50.4)	.63
Fulvestrant	269 (25.3)	28 (23.9)	241 (25.4)	
Aromatase inhibitor	262 (24.6)	33 (28.2)	229 (24.2)	
Treatment line				
1	313 (32.9)	34 (32.1)	279 (33.0)	.61
2	200 (21.0)	19 (17.9)	181 (21.4)	
≥3	438 (46.1)	53 (50.0)	385 (45.6)	

^a^ Percentages are calculated from the row totals.

**Table 2.** Characteristics of the Cohort in the Hormone Receptor–Positive/ERBB2-Negative Subpopulation by Race

Characteristic	No. (%) of patients^a^			Fisher test *P* value
	Overall (N = 820)	Black (n = 89)	White (n = 731)	
Progesterone receptor				
Positive	530 (65.8)	62 (71.3)	468 (65.2)	.28
Negative	275 (34.2)	25 (28.7)	250 (34.8)	
Histotype				
Ductal	536 (77.3)	60 (83.3)	476 (76.6)	.44
Lobular	116 (16.7)	10 (13.9)	106 (17.1)	
Mixed	41 (5.9)	2 (2.8)	39 (6.3)	
Liver metastases				
No	516 (63.0)	55 (61.8)	461 (63.2)	.82
Yes	303 (37.0)	34 (38.2)	269 36.8)	
Lung metastases				
No	596 (72.8)	58 (65.2)	538 (73.8)	.10
Yes	223 (27.2)	31 (34.8)	192 (26.3)	
Central nervous system metastases				
No	764 (94.2)	81 (91.0)	683 (94.6)	.22
Yes	47 (5.8)	8 (9.0)	39 (5.4)	
Bone metastases				
No	210 (25.6)	24 (27.0)	186 (25.5)	.80
Yes	609 (74.4)	65 (73.0)	544 (74.5)	
Nodal metastases				
No	537 (65.6)	53 (59.6)	484 (66.3)	.24
Yes	282 (34.4)	36 (40.4)	246 (33.7)	
Skin or soft tissue metastases				
No	698 (85.2)	78 (87.6)	620 (84.9)	.63
Yes	121 (14.8)	11 (12.4)	110 (15.1)	
De novo metastatic breast cancer				
No	551 (77.3)	53 (69.7)	498 (78.2)	.11
Yes	162 (22.7)	23 (30.3)	139 (21.8)	
Endocrine therapy type				
None	262 (36.0)	26 (33.8)	236 (36.3)	.84
Fulvestrant	240 (33.0)	25 (32.5)	215 (33.1)	
Aromatase inhibitor	225 (31.0)	26 (33.8)	199 (30.6)	
Treatment line				
1	206 (31.0)	22 (29.7)	184 (31.1)	.95
2	150 (22.6)	16 (21.6)	134 (22.7)	
≥3	309 (46.5)	36 (48.6)	273 (46.2)	

^a^ Percentages are calculated from the row totals.

### Racial Differences in Genetic Alterations Detected by ctDNA

**Figure 1.**
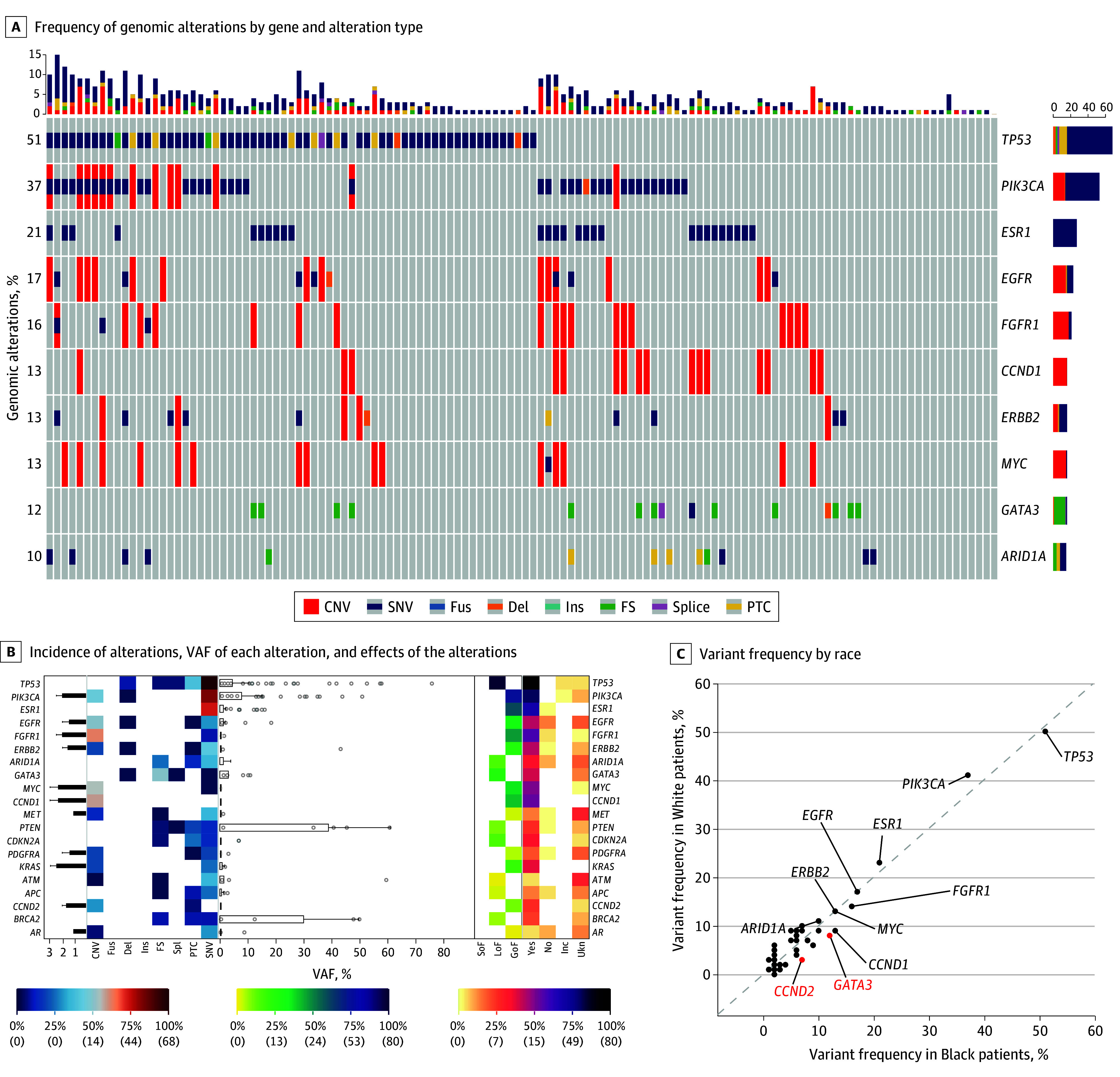
Genomic Landscape of Black Patients With Metastatic Breast Cancer in the Cohort In panel B, the color scale bars represent variant frequency in both percentage and number (in parentheses), and error bars indicate 95% CIs. In panel C, red indicates *P* < .05. CNV indicates copy number variant; Del, deletion; FS, frameshift; Fus, fusion; GoF, gain of function; Inc, inconclusive; Ins, insertion; LoF, loss of function; PTC, premature termination codon; SNV, single-nucleotide variant; SoF, switch of function; Spl, splicing variance; Ukn, unknown; VAF, variant allelic frequency.

We found no significant associations in clinical variables between Black and White patients (eTable 1 in Supplement 1). For ctDNA results, by logistic regression, Black patients in the cohort had higher rates of *CDKN2A* (OMIM 600160) SNV (OR, 5.37; 95% CI, 1.49-19.27; *P* = .01), *GATA3* (OMIM 131320) SNV (OR, 1.99; 95% CI, 1.05-3.75; *P* = .03), *PTPN11* (OMIM 176876) SNV (OR, 7.96; 95% CI, 1.11-56.99; *P* = .04), and *CCND2* (OMIM 123833) CNV (OR, 3.36; 95% CI, 1.37-8.25; *P* = .008) (eTable 2 in Supplement 1). On multivariate analysis, Black patients had a significantly higher rate of *GATA3* SNV (OR, 2.31; 95% CI, 1.17-4.54; *P* = .02) and *CCND2* CNV (OR, 4.63; 95% CI, 1.79-11.97; *P* = .002) (Figure 1; eTable 3 in Supplement 1). After variable selection in the univariate analysis, there were no pathway associations on multivariate analysis (eTable 4 in Supplement 1). There was no significant difference in VAF between Black and White patients.

There were no significant associations observed for clinical variables in the HR^+^/ERBB2^−^ population (eTable 5 in Supplement 1). In this subpopulation, using logistic regression, self-reported Black race was associated with *CDKN2A* SNV (OR, 5.07; 95% CI, 1.19-21.57; *P* = .03), *GATA3* SNV (OR, 2.22; 95% CI, 1.13-4.36; *P* = .02), *PTPN11* SNV (OR, 16.78; 95% CI, 1.51-186.98; *P* = .02), *KIT* (OMIM 164920) CNV (OR, 4.22; 95% CI, 1.03-17.16; *P* = .04), *PDGFRA* (OMIM 173490) CNV (OR, 3.90; 95% CI, 1.32-11.48; *P* = .01), *CCND1* (OMIM 168461) CNV (OR, 1.91; 95% CI, 1.02-3.57; *P* = .04), and *CCND2* CNV (OR, 4.21; 95% CI, 1.03-17.16; *P* = .04) (eTable 6 in Supplement 1). On multivariate analysis, Black patients had a significantly higher rate of *GATA3* SNV (OR, 2.04; 95% CI, 1.02-4.80; *P* = .04) and *PDGFRA* CNV (OR, 3.95; 95% CI, 1.33-11.72; *P* = .01) (eTable 7 in Supplement 1). There were no pathway associations on univariate analysis (eTable 8 in Supplement 1).

We plotted the specific alterations in *GATA3* in the cohort for both Black and White patients, and the most common *GATA3* alteration in both subgroups was P400fs. We then plotted the specific alterations in *PIK3CA* for both racial subgroups, as this has therapeutic impact, and the most common alteration location was H1047R/L (eFigure in Supplement 1). There were no statistically significant differences in the frequency of hotspot alterations.

### Oncogenic Pathway Variants by Race

The overall landscape of genetic pathway variants in Black and White patients within the cohort was assessed. Black patients most commonly had alterations in the pathways of p53 SNV (64 of 135 [47.4%]), PI3K SNV (43 of 135 [31.8%]), receptor tyrosine kinase CNV (37 of 135 [27.4%]), estrogen receptor (ER) SNV (36 of 135 [26.7%]), MYC CNV (15 of 135 [11.1%]), and RAF CNV (12 of 135 [8.9%]) (eTable 9 in Supplement 1). None of the Black patients represented in the cohort had mutations in the MEK or NRF2 pathways. The White patients in the cohort most frequently had mutations in the pathways of p53 SNV (455 of 1061 [42.9%]), PI3K SNV (364 of 1061 [34.3%]), ER SNV (243 of 1061 [22.9%]), receptor tyrosine kinase CNV (221 of 1061 [20.8%]), MYC CNV (98 of 1061 [9.2%]), and PI3K CNV (97 of 1061 [9.1%]) (eTable 9 in Supplement 1).

### Racial Differences in Genetic Alterations Detected by ctDNA

**Figure 2.**
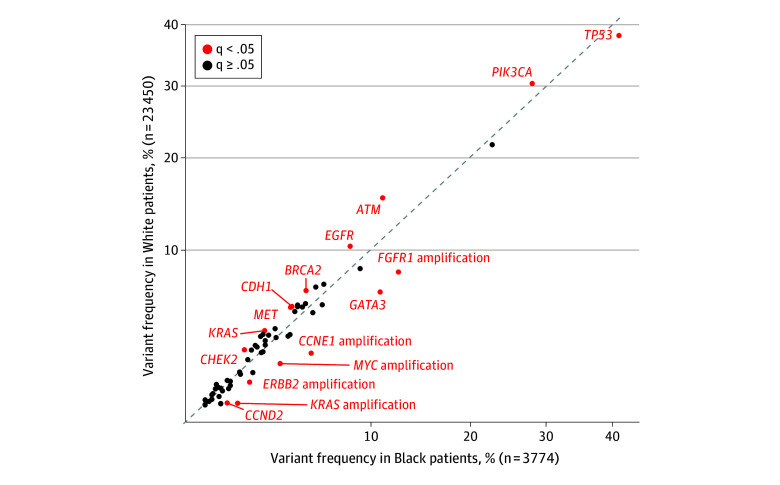
Comparison of Variant Frequency by Race in the GuardantINFORM Database

Using the GuardantINFORM database to validate our findings, we found differences in somatic alterations detected by ctDNA between Black and White patients with mBC. Black patients had significantly higher frequencies of *TP53* (OMIM 191170) (41.3% vs 38.1% in White patients), *GATA3* (10.9% vs 6.7%), and *CCND2* (1.7% vs 1.1%) alterations, as well as higher rates of *FGFR1* (OMIM 136350) (12.5% vs 8.2%), *CCNE1* (OMIM 123837) (5.7% vs 3.0%), *MYC* (OMIM 190080) (3.9% vs 2.5%), *ERBB2* (OMIM 164870) (2.5% vs 1.7%), and *KRAS* (OMIM 190070) amplifications (2.0% vs 1.0%) when compared with White patients (q < 0.05). White patients had significantly higher frequencies of *PIK3CA* (30.3% vs 27.9%), *ATM* (OMIM 607580) (15.2% vs 11.1%), *EGFR* (OMIM 131550) (10.4% vs 8.4%), *BRCA2* (OMIM 600185) (6.8% vs 5.4%), *CDH1* (OMIM 192090) (5.7% vs 4.6%), *MET* (OMIM 164860) (5.6% vs 4.5%), *KRAS* (OMIM 190070) (4.2% vs 3.2%), and *CHEK2* (OMIM 604373) (3.1% vs 2.3%) alterations (q < 0.05) (Figure 2).

### Targeted Treatment Analysis

Of those patients with *PIK3CA* SNVs in our cohort, Black patients were significantly less likely to receive targeted therapy with PI3K inhibitors than White patients (1 of 17 [5.9%] vs 45 of 156 [28.8%], Fisher exact test *P* = .04) (eTable 10 in Supplement 1). However, there was no difference in the use of CDK4/6 or mTOR inhibitors, which do not require specific alterations for therapy initiation (eTables 11 and 12 in Supplement 1). Among patients with *PIK3CA* SNVs, none of the Black patients were enrolled in a clinical trial vs 11.5% of the White patients. There was a significant difference in median HbA_1c_ level between Black and White patients with *PIK3CA* mutations (6.1% vs 5.5%; *P* = .01). There was a numerical but not statistically significant difference between Black and White patients with HbA_1c_ levels greater than 6.4% (5 of 17 [29.4%] vs 18 of 117 [15.4%]) (χ^2^ test *P* = .15) (eTable 13 in Supplement 1).

### Prognostic Analysis

After variable selection in the univariate analysis (eTables 14-19 in Supplement 1), Black patients had a significantly worse prognosis with liver metastases (hazard ratio [HR], 2.21; 95% CI, 1.25-3.92; *P* = .007) and *MYC* CNV (HR, 3.97; 95% CI, 1.76-8.97; *P* = .001) (eTable 20 in Supplement 1). There were no prognostic associations with genetic pathway variants in the multivariate analysis (eTable 21 in Supplement 1). Black patients in the HR^+^/ERBB2^−^ population had significantly worse prognosis with alterations in the *PI3K* SNV pathway (OR, 2.19; 95% CI, 1.14-4.21; *P* = .02) but had no other prognostic associations on multivariate analysis (eTable 22 in Supplement 1).

After the selection of variables in the univariate analysis (eTables 23-28 in Supplement 1), White patients had numerous prognostic associations on multivariate analysis (eTable 29 in Supplement 1), including liver metastases (HR, 1.49; 95% CI, 1.21-1.83; *P* < .001), soft tissue metastases (HR, 1.52; 95% CI, 1.18-1.96; *P* = .001), central nervous system metastases (HR, 1.47; 95% CI, 1.03-2.11; *P* = .03), *TP53* SNV (HR, 1.79; 95% CI, 1.46-2.19; *P* < .001), *ESR1* (OMIM 133430) SNV (HR, 1.39; 95% CI, 1.10-1.76; *P* = .007), and *MYC* CNV (HR, 1.39; 95% CI, 1.03-2.16; *P* = .03). These findings remained significant in the White HR^+^/ERBB2^−^ population (Table 2; eTable 30 in Supplement 1). On multivariate analysis, we found a negative prognostic association with pathway alterations for *p53* SNVs (HR, 1.73; 95% CI, 1.42-2.12; *P* < .001), *RAF* SNVs (HR, 1.65; 95% CI, 1.08-2.54; *P* = .02), and *PI3K* CNVs (HR, 1.52; 95% CI, 1.07-2.15; *P* = .02) (eTable 31 in Supplement 1). Considering the White HR^+^/ERBB2^−^ subgroup, we found a negative prognostic association on multivariate analysis for *p53* SNVs (HR, 1.47; 95% CI, 1.16-1.86; *P* < .001), *MYC* CNVs (HR, 1.93; 95% CI, 1.24-2.99; *P* = .003), and *PI3K* CNVs (HR, 1.60; 95% CI, 1.04-2.45; *P* = .03) (eTable 32 in Supplement 1).

### Survival Analysis

**Figure 3.**
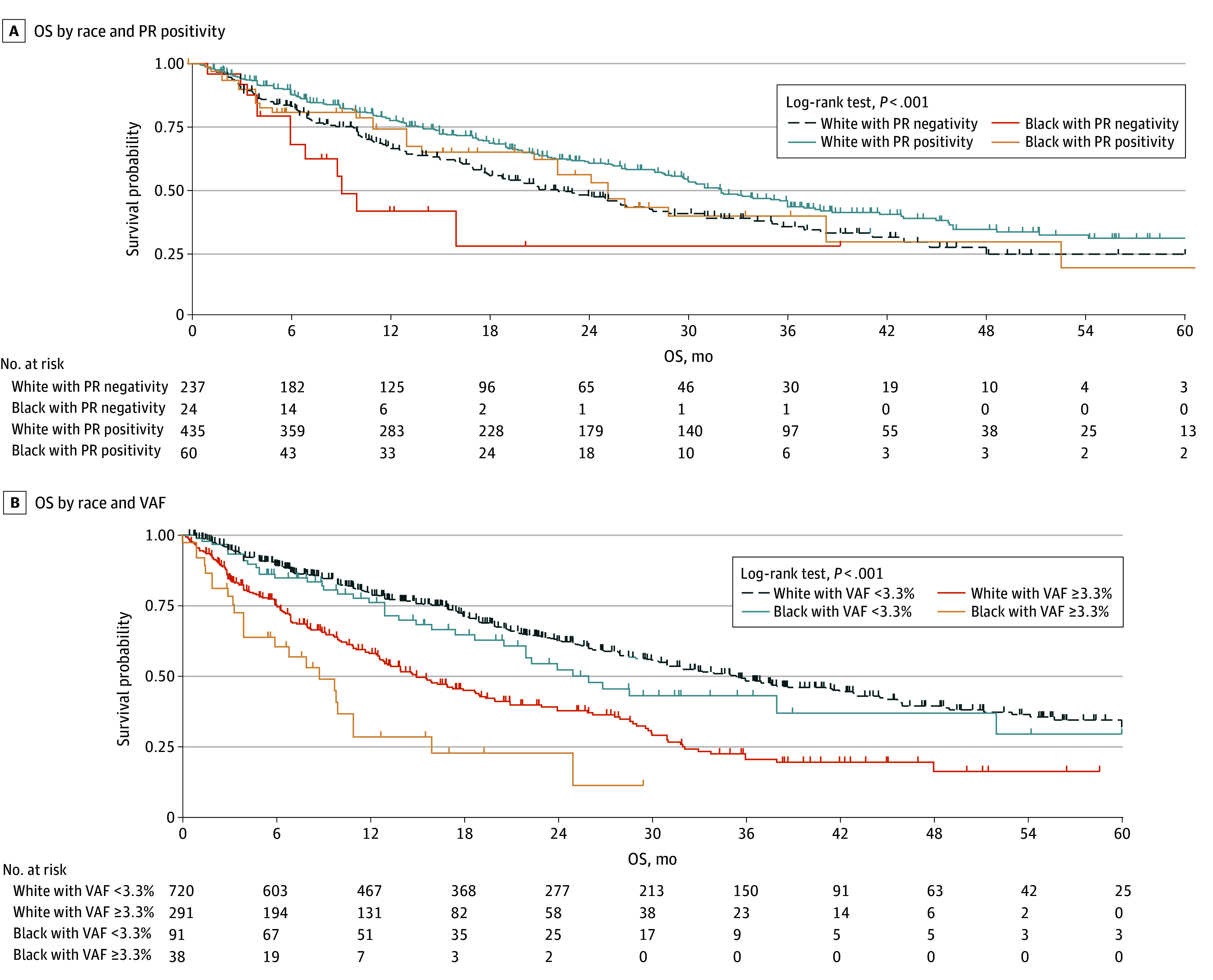
Kaplan-Meier Plots for Overall Survival (OS) by Race and Variant Allelic Frequency (VAF) and Race and Progesterone Receptor (PR) Positivity in Patients With Hormone Receptor–Positive and ERBB2-Negative Metastatic Breast Cancer.

Black patients had a significantly shorter OS than White patients (median [IQR] OS, 22.0 [8.1-52.0] vs 29.0 [11.2-61.0] months; log-rank test *P* = 5.58 × 10^−22^). Patients with a higher VAF had shorter OS than those with lower VAF, and the shortest median overall OS was seen in Black patients with VAFs of 3.3% or higher.^33^ We then determined OS in the ER^+^/progesterone receptor–positive [PR^+^]/ERBB2^−^ and ER^+^/PR^−^/ERBB2^−^groups. The median (IQR) OS of Black patients with ER^+^/PR^+^/ERBB2^−^ disease was 25.0 (11.0-52.0) months vs 32.0 (13.7-61.0) months for White patients. The median (IQR) OS of Black patients with ER^+^/PR^−^/ERBB2^−^ tumors was 9.1 (6.0 to not applicable) months vs 21.0 (8.7-48.0) months for White patients. The difference in OS among these groups was significant (log-rank test *P* < .001) (Figure 3).

## Discussion

We detail here our key findings of racial differences in ctDNA profiles, treatment inequities, and OS disparities in patients with mBC. First, our study revealed that Black patients had higher frequencies of *GATA3* SNV and *CCND2* CNV on ctDNA genomic profiling by multivariate analysis, validated in a large, population-based evidence database. Prior studies evaluating ctDNA profiles in patients with mBC have been restricted in their ability to determine racial differences by small sample sizes with limited numbers of Black patients.^34,35,36,37,38^ GATA3 is a transcription factor involved in the differentiation of breast epithelial cells, and *GATA3* alterations have been found in approximately 10% of breast cancers.^39,40^ Patients with lower levels of intact GATA3 have poorly differentiated tumors, larger tumor size, and higher histologic grades.^41,42^ There is no current treatment targeting *GATA3* alterations. *CCND2* is a gene that encodes protein G1/S-specific cyclin D2, which regulates CDK4/6 in the cell cycle. Dysregulation of this process through *CCND2* alteration can cause tumor proliferation and cell growth. *CCND2* is currently under investigation as a potential drug target for breast cancer treatment.^43,44,45^

A second key finding of this study was that Black patients had a poor prognostic association with *MYC* CNV in the overall cohort and alterations in the *PIK3CA* SNV pathway in the HR^+^/ERBB2^−^ population. There is evidence that *MYC* alterations may drive endocrine therapy resistance, which could potentially explain the poor prognostic association.^46^ The *PIK3CA* SNV pathway contains actionable alterations, and the poor prognostic association may be due to a lack of access to targeted treatments given the small difference in detecting *PIK3CA* alterations between Black and White patients.

A third key finding in our study is that, even in patients with a targetable alteration in *PIK3CA* in our cohort, Black patients were less likely to receive targeted therapy and be enrolled in a clinical trial when compared with White patients. No differences were observed in use of CDK4/6 or mTOR inhibitors that do not require detection of specific alterations for therapy initiation. There are several potential reasons for this disparity. PI3K inhibitors can cause hyperglycemia, and physicians may be hesitant to prescribe them in patients with elevated HbA_1c_ or elevated fasting glucose levels. Black patients have been found to have a higher mean HbA_1c_ level than White patients despite no difference in mean blood glucose level.^47,48,49,50^ In our cohort, Black patients had a significantly higher median HbA_1c_ level, potentially leading to exclusion from clinical trial enrollment and targeted treatment use. Another reason for the finding may be the cost of the medication. In the US, Black patients are less likely to be insured compared with White patients.^51,52^ Determining insurance status in our cohort will be a point of further investigation, because this may be influencing access to or offers of targeted treatments. Our finding that Black patients with *PIK3CA* alterations were less likely to be enrolled in clinical trials compared with White patients is aligned with public health data that Black patients are underrepresented in cancer clinical trials.^8,9,10,11,12,53,54,55,56,57,58^ This is an area of increasing concern within oncology, and efforts to diversify clinical trials must continue, including ongoing research to identify barriers, promoters, and solutions for equitable clinical trial participation.^10,53,59,60,61,62,63,64^

Fourth, we found shorter OS from first ctDNA test in Black patients compared with White patients. This finding aligns with prior studies and epidemiological data.^1,5,6,7,65,66,67,68,69,70^ Patients with higher VAF had shorter OS, and Black patients with higher VAF had the poorest outcomes. Higher ctDNA VAF is associated with shorter progression-free survival and OS, and adding VAF to a prognostic model of mBC helped with model performance.^71^ The separation of the Kaplan-Meier curves occurs early, indicating that there are likely external factors impacting prognosis, such as social determinants of health. Further research to evaluate social determinants of health and their impact on ctDNA testing, ability to attend clinic appointments, treatment adherence, treatment type, and overall outcomes should be performed.

### Limitations

Our study has several limitations. It is a retrospective cohort study, and comorbidities, lifestyle factors, and social determinants of health were not fully accounted for in the analyses. The study may have selection bias because the patients in this study had access to ctDNA testing at large, urban academic centers and opted into receiving it. The number of patients enrolled in clinical trials was small in our cohort, and it may be challenging to draw strong conclusions from these data. Self-reported race in this study may not accurately reflect genetic ancestry, which could provide a more precise understanding of the somatic tumor variations observed.^72^

## Conclusions

In this cohort study, we found that Black patients with mBC had somatic genomic differences on ctDNA analysis compared with White patients, but these findings were unlikely to account for differences in equitable use of targeted treatments and shorter OS in Black patients. Future preclinical studies should assess the impact of these observed somatic differences on the biology of mBC, and clinical studies should further evaluate how HbA_1c_ and social determinants of health may impact clinical trial enrollment and OS. The disparity in use of targeted treatments for patients with *PIK3CA* alterations shows that clinical inequities exist alongside genomic differences, which must be a focus for future implementation of science interventions.

## Notes

Supplement 1. **eFigure.** Lollipop plots demonstrating the distribution of genomic alterations by their amino acid coordinates in (A) GATA3 in Black patients with mBC, (B) GATA3 in White patients with mBC, (C) PIK3CA in Black patients with mBC, and (D) PIK3CA in White patients with mBC **eTable 1.** Univariate logistic association of clinical characteristics of the population (Black vs. White) **eTable 2.** Univariate logistic association of single gene alterations in the general population (Black versus White). **eTable 3.** Multivariate logistic association in the general population (Black versus White) **eTable 4.** Univariate logistic association of pathway alterations in the general population (Black versus White) **eTable 5.** Univariate logistic association of clinical characteristics in the HR+/HER2- population (Black versus White) **eTable 6.** Univariate logistic association of single genes alterations in the HR+/HER2- population (Black versus White) **eTable 7.** Multivariate logistic association of clinical characteristics and gene alterations in the HR+/HER2- population (Black versus White) **eTable 8.** Univariate logistic association of pathway alterations in the HR+/HER2- population (Black versus White) **eTable 9.** Distribution of pathway alterations in the general population and in the two subgroups (White versus Black **eTable 10.** Number of patients who received targeted therapy for PI3K in the White and Black populations **eTable 11.** Number of patients who received a CDK 4/6 inhibitor in the White and Black populations **eTable 12.** Number of patients who received an mTOR inhibitor in the White and Black populations **eTable 13.** Differences in A1c between Black and White patients with *PIK3CA* mutations **eTable 14.** Univariate Cox regression analysis of clinical characteristics in the Black population **eTable 15.** Univariate Cox regression analysis of single gene alterations in the Black population **eTable 16.** Univariate Cox regression analysis of pathway alterations in the Black population **eTable 17.** Univariate Cox regression analysis of clinical characteristics in the HR+/HER2- Black population. **eTable 18.** Univariate Cox regression analysis of single gene alterations in the HR+/HER2- Black population. **eTable 19.** Univariate Cox regression analysis of pathway alterations in the HR+/HER2- Black population **eTable 20.** Multivariate Cox regression analysis of clinical characteristics and single gene alterations in the Black population **eTable 21.** Multivariate Cox regression analysis of clinical characteristics and pathway alterations in the Black population **eTable 22.** Multivariate Cox regression analysis of clinical characteristics and pathway alterations in the HR+/HER2- Black population **eTable 23.** Univariate Cox regression analysis of clinical characteristics in the White population **eTable 24.** Univariate Cox regression analysis of single gene alterations in the White population **eTable 25.** Univariate Cox regression analysis of pathways alterations in the White population **eTable 26.** Univariate Cox regression analysis of clinical characteristics in the HR+/HER2- White population **eTable 27.** Univariate Cox regression analysis of single gene alterations in the HR+/HER2- White population **eTable 28.** Univariate Cox regression analysis of pathway alterations in the HR+/HER2- White population **eTable 29.** Multivariate Cox regression analysis of clinical characteristics and single gene alterations in the White population **eTable 30.** Multivariate Cox regression analysis of clinical characteristics and single gene alterations in the HR+/HER2- White population **eTable 31.** Multivariate Cox regression analysis of clinical characteristics and pathway alterations in the White population **eTable 32.** Multivariate Cox regression analysis of clinical characteristics and pathway alterations in the HR+/HER2- White population

Supplement 2.
Data Sharing Statement
