# Error in Affiliations

**DOI:** 10.1001/jamanetworkopen.2025.11673

**Published:** 2025-04-21

In the Original Investigation titled “Racial Differences in ctDNA Profiles, Targeted Therapy Use, and Outcomes in Metastatic Breast Cancer,”^1^ published February 26, 2025, an affiliation (Department of Medical Oncology, CRO Aviano, National Cancer Institute, IRCCS, Aviano, Italy) was missing and has been added for authors Lorenzo Foffano, Lorenzo Gerratana, and Fabio Puglisi. Additionally, for the affiliation the Department of Medicine, University of Udine, the city was incorrectly listed as Aviano; the correct city is Udine. This article has been corrected.^1^
