# Supplementary material for: Racial Differences in ctDNA Profiles, Targeted Therapy Use, and Outcomes in Metastatic Breast Cancer
Source: JAMA Netw Open. 2025 Feb 26;8(2):e2461899. doi: 10.1001/jamanetworkopen.2024.61899 (PMC11866032; doi:10.1001/jamanetworkopen.2024.61899)
Supplement: Supplement 1. — eFigure. Lollipop plots demonstrating the distribution of genomic alterations by their amino acid coordinates in (A) GATA3 in Black patients with mBC, (B) GATA3 in White patients with mBC, (C) PIK3CA in Black patients with mBC, and (D) PIK3CA in White patients with mBC eTable 1. Univariate logistic association of clinical characteristics of the population (Black vs. White) eTable 2. Univariate logistic association of single gene alterations in the general population (Black versus White). eTable 3. Multivariate logistic association in the general population (Black versus White) eTable 4. Univariate logistic association of pathway alterations in the general population (Black versus White) eTable 5. Univariate logistic association of clinical characteristics in the HR+/HER2- population (Black versus White) eTable 6. Univariate logistic association of single genes alterations in the HR+/HER2- population (Black versus White) eTable 7. Multivariate logistic association of clinical characteristics and gene alterations in the HR+/HER2- population (Black versus White) eTable 8. Univariate logistic association of pathway alterations in the HR+/HER2- population (Black versus White) eTable 9. Distribution of pathway alterations in the general population and in the two subgroups (White versus Black eTable 10. Number of patients who received targeted therapy for PI3K in the White and Black populations eTable 11. Number of patients who received a CDK 4/6 inhibitor in the White and Black populations eTable 12. Number of patients who received an mTOR inhibitor in the White and Black populations eTable 13. Differences in A1c between Black and White patients with PIK3CA mutations eTable 14. Univariate Cox regression analysis of clinical characteristics in the Black population eTable 15. Univariate Cox regression analysis of single gene alterations in the Black population eTable 16. Univariate Cox regression analysis of pathway alterations in the Black population eTable 17. Univariate [file jamanetwopen-e2461899-s001.pdf]

Supplemental Online Content

Podany EL, Foffano L, Gerratana L, et al. Racial differences in ctDNA profiles, targeted therapy use, and outcomes in metastatic breast cancer. *JAMA Netw Open*. 2025;8(2):e2461899. doi:10.1001/jamanetworkopen.2024.61899

eFigure. Lollipop plots demonstrating the distribution of genomic alterations by their amino acid coordinates in (A) GATA3 in Black patients with mBC, (B) GATA3 in White patients with mBC, (C) PIK3CA in Black patients with mBC, and (D) PIK3CA in White patients with mBC

eTable 1. Univariate logistic association of clinical characteristics of the population (Black vs. White)

eTable 2. Univariate logistic association of single gene alterations in the general population (Black versus White).

eTable 3. Multivariate logistic association in the general population (Black versus White)

eTable 4. Univariate logistic association of pathway alterations in the general population (Black versus White)

eTable 5. Univariate logistic association of clinical characteristics in the HR+/HER2- population (Black versus White)

eTable 6. Univariate logistic association of single genes alterations in the HR+/HER2- population (Black versus White)

eTable 7. Multivariate logistic association of clinical characteristics and gene alterations in the HR+/HER2- population (Black versus White)

eTable 8. Univariate logistic association of pathway alterations in the HR+/HER2- population (Black versus White)

eTable 9. Distribution of pathway alterations in the general population and in the two subgroups (White versus Black

eTable 10. Number of patients who received targeted therapy for PI3K in the White and Black populations

eTable 11. Number of patients who received a CDK 4/6 inhibitor in the White and Black populations

eTable 12. Number of patients who received an mTOR inhibitor in the White and Black populations

eTable 13. Differences in A1c between Black and White patients with *PIK3CA* mutations

eTable 14. Univariate Cox regression analysis of clinical characteristics in the Black population

eTable 15. Univariate Cox regression analysis of single gene alterations in the Black population

© 2025 Podany EL et al. *JAMA Network Open*.

eTable 16. Univariate Cox regression analysis of pathway alterations in the Black population

eTable 17. Univariate Cox regression analysis of clinical characteristics in the HR+/HER2- Black population.

eTable 18. Univariate Cox regression analysis of single gene alterations in the HR+/HER2- Black population.

eTable 19. Univariate Cox regression analysis of pathway alterations in the HR+/HER2- Black population

eTable 20. Multivariate Cox regression analysis of clinical characteristics and single gene alterations in the Black population

eTable 21. Multivariate Cox regression analysis of clinical characteristics and pathway alterations in the Black population

eTable 22. Multivariate Cox regression analysis of clinical characteristics and pathway alterations in the HR+/HER2- Black population

eTable 23. Univariate Cox regression analysis of clinical characteristics in the White population

eTable 24. Univariate Cox regression analysis of single gene alterations in the White population

eTable 25. Univariate Cox regression analysis of pathways alterations in the White population

eTable 26. Univariate Cox regression analysis of clinical characteristics in the HR+/HER2- White population

eTable 27. Univariate Cox regression analysis of single gene alterations in the HR+/HER2- White population

eTable 28. Univariate Cox regression analysis of pathway alterations in the HR+/HER2- White population

eTable 29. Multivariate Cox regression analysis of clinical characteristics and single gene alterations in the White population

eTable 30. Multivariate Cox regression analysis of clinical characteristics and single gene alterations in the HR+/HER2- White population

eTable 31. Multivariate Cox regression analysis of clinical characteristics and pathway alterations in the White population

eTable 32. Multivariate Cox regression analysis of clinical characteristics and pathway alterations in the HR+/HER2- White population

This supplemental material has been provided by the authors to give readers additional information about their work.

eFigure 1. Lollipop plots demonstrating the distribution of genomic alterations by their amino acid coordinates in (A) GATA3 in Black patients with mBC, (B) GATA3 in White patients with mBC, (C) PIK3CA in Black patients with mBC, and (D) PIK3CA in White patients with mBC.

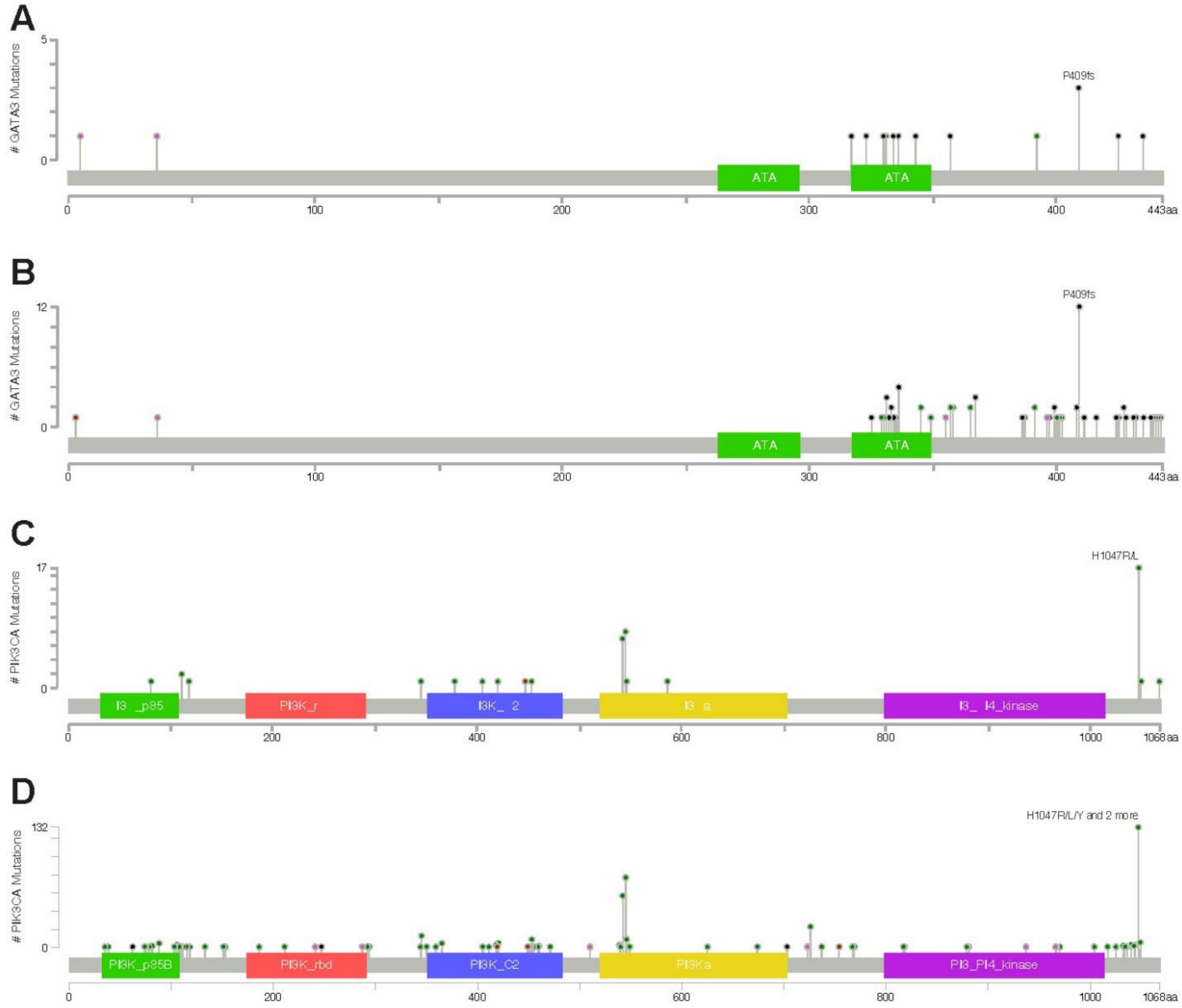

© 2025 Podany EL et al. *JAMA Network Open*.

eTable 1. Univariate logistic association of clinical characteristics of the population (Black vs. White).

Characteristic	OR	95% C.I.	P>|z|
**Lung**	1.28	0.87-1.87	0.21
**Liver**	1.06	0.73-1.53	0.77
**Bone**	0.87	0.60-1.27	0.47
**Node**	1.19	0.83-1-71	0.35
**Soft tissue**	1.05	0.66-1.67	0.83
**CNS**	1.46	0.81-2.61	0.20
**De novo**	1.39	0.90-2.15	0.13
**ET type**			
Fulvestrant	0.99	0.61-1.60	0.97
AI	1.23	0.78-1.94	0.38
**Treatment line**			
2	0.86	0.48-1.56	0.627
>=3	1.13	0.71-1.78	<0.001

Abbreviations: OR, Odds Ratio; C.I., Confidence Interval; ET, Endocrine Therapy; CNS, central nervous system

© 2025 Podany EL et al. *JAMA Network Open*.

eTable 2. Univariate logistic association of single gene alterations in the general population (Black versus White).

Alteration	OR	95% C.I.	P>|z|
**PIK3CA SNVs**	0.96	0.64-1.43	0.83
**ESR1 SNVs**	1.03	0.66 – 1.61	0.89
**TP53 SNVs**	1.24	0.87-1.78	0.24
**ARID1A SNVs**	0.88	0.37-2.10	0.78
**ERBB2 SNVs**	1.40	0.61-3.18	0.43
**CDKN2A SNVs**	5.37	1.49-19.27	0.01
**NF1 SNVs**	0.26	0.35-1.90	0.18
**PTEN SNVs**	1.00	0.42-2.39	0.99
**CDH1 SNVs**	0.60	0.14-2.55	0.49
**AKT1 SNVs**	1.16	0.45-3.02	0.76
**GATA3 SNVs**	1.99	1.05-3.75	0.03
**SMAD4 SNVs**	0.32	0.04-2.40	0.27
**RB1 SNVs**	0.19	0.03-1.40	0.10
**FBXW7 SNVs**	1.97	0.22-17.78	0.54
**HRAS SNVs**	0.87	0.11-6.94	0.90
**GNAS SNVs**	2.00	0.74-5.42	0.17
**BRCA2 SNVs**	1.66	0.67-4.06	0.27
**KRAS SNVs**	1.60	0.65-3.91	0.30
**NRAS SNVs**	1.58	0.18-13.59	0.68
**BRCA1 SNVs**	0.74	0.17-3.21	0.69
**VHL SNVs**	7.91	0.49-127.21	0.14
**BRAF SNVs**	0.35	0.047-2.63	0.31
**APC SNVs**	1.77	0.59-5.31	0.31
**JAK2 SNVs**	3.95	0.36-43.87	0.26
**RAF1 SNVs**	2.63	0.27-25.48	0.40

© 2025 Podany EL et al. *JAMA Network Open*.

	OR	95% C.I.	P>|z|
**STK11 SNVs**	0.78	0.09-6.18	0.82
**PTPN11 SNVs**	7.96	1.11-56.99	0.04
**ATM SNVs**	0.20	0.03-1.44	0.11
**NOTCH1 SNVs**	3.95	0.36-43.87	0.26
**HRAS SNVs**	0.87	0.11-6.94	0.90
**RAF1 SNVs**	2.63	0.27-25.48	0.40
**KIT CNVs**	1.51	0.51-4.47	0.45
**PDGFRA CNVs**	1.53	0.58-4.06	0.39
**BRAF CNVs**	1.22	0.57-2.63	0.61
**ERBB2 CNVs**	0.91	0.35-2.34	0.85
**CCND1 CNVs**	1.65	0.93-2.91	0.08
**KRAS CNVs**	1.24	0.47-3.23	0.66
**AR CNVs**	1.05	0.24-4.64	0.95
**RAF1 CNVs**	0.98	0.34-2.82	0.97
**CDK6 CNVs**	0.57	0.17-1.85	0.35
**PIK3CA CNVs**	1.06	0.58-1.95	0.85
**EGFR CNVs**	1.28	0.71-2.32	0.42
**MYC CNVs**	1.23	0.69-2.18	0.48
**MET CNVs**	1.13	0.38-3.26	0.83
**CCNE1 CNVs**	0.76	0.32-1.80	0.54
**CCND2 CNVs**	3.36	1.37-8.25	0.008
**FGFR2 CNVs**	0.56	0.07-4.28	0.57

Abbreviations: SNVs, Single Nucleotide Variants; CNVs, Copy number variants; OR, Odds Ratio; C.I., Confidence Interval; ET, Endocrine Therapy

© 2025 Podany EL et al. *JAMA Network Open*.

eTable 3. Multivariate logistic association in the general population (Black versus White).

Alteration	OR	95% C.I.		P>|z|
**GATA3 SNVs**				
mut	2.31	1.17	4.54	0.02
**CCND2 CNVs**				
ampl	4.63	1.79	11.97	0.002
**Characteristic**				
**Treatment line**				
2	0.84	0.46	1.53	0.57
3	1.10	0.69	1.75	0.68

Abbreviations: SNVs, Single Nucleotide Variants; CNVs, Copy number variants; OR, Odds Ratio; C.I., Confidence Interval

© 2025 Podany EL et al. *JAMA Network Open*.

eTable 4. Univariate logistic association of pathway alterations in the general population (Black versus White).

Alteration	OR	95% C.I.	P>|z|
**PI3K SNVs**	0.89	0.61-1.31	0.57
**ER SNVs**	1.22	0.81-1.84	0.33
**P53 SNVs**	1.20	0.84-1.71	0.32
**RTK SNVs**	0.83	0.39-1.76	0.62
**Cell Cycle SNVs**	0.83	0.32-2.12	0.70
**RAS SNVs**	1.12	0.56-2.22	0.75
**NOTCH SNVs**	2.64	0.53-13.23	0.24
**RAF SNVs**	0.58	0.14-2.45	0.45
**WNT SNVs**	1.59	0.54-4.72	0.40
**RTK CNVs**	1.44	0.96-2.15	0.08
**RAF CNVs**	1.28	0.68-2.43	0.44
**Cell Cycle CNVs**	1.24	0.79-1.96	0.35
**RAS CNVs**	1.24	0.47-3.23	0.66
**ER CNVs**	0.92	0.21-4.04	0.92
**PI3K CNVs**	1.06	0.58-1.95	0.85
**MYC CNVs**	1.23	0.69-2.18	0.48

Abbreviations: SNVs, Single Nucleotide Variants; CNVs, Copy number variants; OR, Odds Ratio; C.I., Confidence Interval; ET, Endocrine Therapy

© 2025 Podany EL et al. *JAMA Network Open*.

eTable 5. Univariate logistic association of clinical characteristics in the HR+/HER2- population (Black versus White).

Characteristic	OR	95% C.I.	P>|z|
**Lung**	1.50	0.94-2.39	0.09
**Liver**	1.06	0.67-1.67	0.80
**Bone**	0.93	0.56-1.52	0.76
**Node**	1.34	0.85-2.10	0.21
**Soft tissue**	0.79	0.41-1.54	0.50
**CNS**	1.73	0.78-3.83	0.18
**De novo**	1.55	0.92-2.63	0.10
**ET type**			
Fulvestrant	1.06	0.59-1.88	0.85
AI	1.19	0.67-2.11	0.56
**Treatment line**			
2	0.99	0.51-1.97	>0.99
>= 3	1.10	0.63-1.94	0.73

Abbreviations: OR, Odds Ratio; C.I., Confidence Interval; ET, Endocrine Therapy; CNS, central nervous system; AI, aromatase inhibitor

© 2025 Podany EL et al. *JAMA Network Open*.

eTable 6. Univariate logistic association of single genes alterations in the HR+/HER2- population (Black versus White).

Alteration	OR	95% C.I.	P>|z|
**PIK3CA SNVs**	1.08	0.68-1.71	0.74
**ESR1 SNVs**	1.16	0.71-1.88	0.55
**TP53 SNVs**	0.96	0.61-1.53	0.87
**ARID1A SNVs**	0.93	0.36-2.41	0.88
**ERBB2 SNVs**	1.55	0.58-4.14	0.38
**CDKN2A SNVs**	5.07	1.19-21.57	0.03
**NF1 SNVs**	0.40	0.05-3.05	0.38
**PTEN SNVs**	1.10	0.38-3.20	0.86
**CDH1 SNVs**	0.71	0.16-3.05	0.64
**AKT1 SNVs**	1.18	0.40-3.45	0.76
**GATA3 SNVs**	2.22	1.13-4.36	0.02
**SMAD4 SNVs**	0.40	0.05-3.05	0.38
**RB1 SNVs**	0.26	0.03-1.90	0.18
**FBXW7 SNVs**	4.14	0.37-46.15	0.25
**GNAS SNVs**	1.03	0.23-4.54	0.97
**BRCA2 SNVs**	0.38	0.05-2.89	0.35
**KRAS SNVs**	1.75	0.65-4.72	0.27
**NRAS SNVs**	4.14	0.37-46.14	0.25
**BRCA1 SNVs**	0.58	0.08-4.48	0.60
**VHL SNVs**	8.30	0.51-133.8	0.14
**BRAF SNVs**	0.48	0.06-3.63	0.47
**APC SNVs**	1.93	0.54-6.90	0.31
**JAK2 SNVs**	4.14	0.37-46.15	0.31
**RAF1 SNVs**	4.14	0.37-46.15	0.25
**PTPN11 SNVs**	16.78	1.51-186.98	0.02

© 2025 Podany EL et al. *JAMA Network Open*.

	OR	95% C.I.	P>|z|
**NOTCH1 SNVs**	8.30	0.51-133.80	0.14
**KIT CNVs**	4.22	1.04-17.16	0.04
**PDGFRA CNVs**	3.90	1.32-11.48	0.01
**BRAF CNVs**	0.68	0.16-2.91	0.60
**CCND1 CNVs**	1.91	1.02-3.57	0.04
**KRAS CNVs**	1.56	0.45-5.46	0.49
**AR CNVs**	1.03	0.13-8.31	0.98
**RAF1 CNVs**	1.31	0.38-4.51	0.67
**PIK3CA CNVs**	0.68	0.26-1.74	0.42
**EGFR CNVs**	1.54	0.73-3.23	0.26
**MYC CNVs**	1.65	0.81-3.38	0.17
**MET CNVs**	1.18	0.26-5.27	0.83
**CCNE1 CNVs**	0.35	0.05-2.62	0.54
**CCND2 CNVs**	4.21	1.03-17.16	0.04

Abbreviations: SNVs, Single Nucleotide Variants; CNVs, Copy number variants; OR, Odds Ratio; C.I., Confidence Interval

© 2025 Podany EL et al. *JAMA Network Open*.

eTable 7. Multivariate logistic association of clinical characteristics and gene alterations in the HR+/HER2- population (Black versus White).

Alteration	OR	95% C.I.		P>|z|
**GATA3 SNV**				
mut	2.04	1.02	4.08	0.04
**PDGFRA CNV**				
ampl	3.95	1.33	11.72	0.01
**CCND1 CNV**				
ampl	1.71	0.90	3.26	0.10

Abbreviations: SNVs, Single Nucleotide Variants; CNVs, Copy number variants; OR, Odds Ratio; C.I., Confidence Interval

© 2025 Podany EL et al. *JAMA Network Open*.

eTable 8. Univariate logistic association of pathway alterations in the HR+/HER2- population (Black versus White).

Alteration	OR	95% C.I.	P>|z|
**PI3K SNVs**	099	0.64-1.56	0.99
**ER SNVs**	1.39	0.88-2.19	0.16
**P53 SNVs**	0.95	0.61-1.51	0.84
**RTK SNVs**	0.87	0.36-2.08	0.76
**Cell Cycle SNVs**	0.88	0.31-2.54	0.82
**RAS SNVs**	1.32	0.60-2.87	0.49
**NOTCH SNVs**	5.58	0.92-33.85	0.06
**RAF SNVs**	0.78	0.18-3.37	0.74
**WNT SNVs**	1.67	0.47-5.87	0.43
**RTK CNVs**	1.60	0.96-2.66	0.07
**RAF CNVs**	1.06	0.41-2.75	0.91
**Cell Cycle CNVs**	1.46	0.82-2.57	0.19
**RAS CNVs**	1.56	0.45-5.46	0.49
**ER CNVs**	0.91	0.11-7.28	0.93
**PI3K CNVs**	0.68	0.26-1.74	0.42
**MYC CNVs**	1.65	0.81-3.38	0.17

Abbreviations: SNVs, Single Nucleotide Variants; CNVs, Copy number variants; OR, Odds Ratio; C.I., Confidence Interval

© 2025 Podany EL et al. *JAMA Network Open*.

eTable 9. Distribution of pathway alterations in the general population and in the two subgroups (White versus Black).

Alteration	Overall patients N(%)	Black patients N(%)	White patients N(%)	p-value
**PI3K SNVs**				
WT	789 (65.97)	92 (68.15)	697 (65.69)	0.57
Mutant	407 (34.03)	43(31.85)	364 (34.31)	
**ER SNVs**				
WT	917 (76.76)	99 (73.33)	818 (77.10)	0.33
Mutant	279 (23.33)	36 (26.67)	243 (22.90)	
**P53 SNVs**				
WT	677 (56.61)	71 (52.59)	606 (57.12)	0.32
Mutant	519 (43.39)	64 (47.41)	455 (42.88)	
**RTK SNVs**				
WT	1113 (93.06)	127 (94.07)	986 (92.93)	0.62
Mutant	83 (6.94)	8 (5.93)	75 (7.07)	
**CELL CYCLE SNVs**				
WT	1144 (95.65)	130 (96.30)	1014 (95.57)	0.70
Mutant	52 (4.35)	5 (3.70)	47 (4.43)	
**RAS SNVs**				
WT	1115 (93.23)	125 (92.59)	990 (93.31)	0.75
Mutant	81 (6.77)	10 (7.41)	71 (6.69)	
**NOTCH SNVs**				
WT	1188 (99.33)	133 (98.52)	1055 (99.43)	0.22
Mutant	8 (0.67)	2 (1.48)	6 (0.57)	
**RAF SNVs**				
WT	1167 (97.58)	133 (98.52)	1034 (97.46)	0.45
Mutant	29 (2.42)	2 (1.48)	127 (2.54)	
**WNT SNVs**				
WT	1172 (97.99)	131 (97.04)	1041 (98.11)	0.40
Mutant	24 (2.01)	4 (2.96)	20 (1.89)	
**MEK SNVs**				
WT	1191 (99.58)	135 (100)	1056 (99.53)	0.42
Mutant	5 (0.42)	0 (0)	5 (0.47)	

© 2025 Podany EL et al. *JAMA Network Open*.

Alteration	Overall patients N(%)	Black patients N(%)	White patients N(%)	p-value
**NRF2 SNVs**				
WT	1191 (99.58)	135 (100)	1056 (99.53)	0.42
Mutant	5 (0.42)	0 (0)	5 (0.47)	
**RAF CNVs**				
WT	1109 (92.73)	123 (91.11)	986 (92.93)	0.44
Mutant	87 (7.27)	12 (8.89)	75 (7.07)	
**CELL CYCLE CNVs**				
WT	999	109 (80.74)	890 (83.88)	0.35
Mutant	197	26 (19.26)	171 (16.12)	
**RAS CNVs**				
WT	1159	130 (96.30)	1029 (96.98)	0.66
Mutant	37	5 (3.70)	32 (3.02)	
**ER CNVs**				
WT	1177	17 (1.60)	1044 (98.40)	0.92
Mutant	19	2 (1.48)	133 (98.52)	
**PI3K CNVs**				
WT	1086	97 (9.14)	964 (90.86)	0.85
Mutant	110	13 (9.63)	122 (90.37)	
**MYC CNVs**				
WT	1083	98 (9.24)	963 (90.76)	0.48
Mutant	113	15 (11.11)	120 (88.89)	

Abbreviations: SNVs, Single Nucleotide Variants; CNVs, Copy number variants; OR, Odds Ratio; C.I., Confidence Interval; WT, Wild type.

© 2025 Podany EL et al. *JAMA Network Open*.

eTable 10. Number of patients who received targeted therapy for PI3K in the White and Black populations.

PI3K therapy	Black N(%)	White N(%)	Total N(%)
**No**	16 (94.12)	111 (71.15)	127 (73.41)
**Yes**	1 (5.88)	45 (28.85)	46 (26.59)
		**Fisher's exact**	0.04

© 2025 Podany EL et al. *JAMA Network Open*.

eTable 11. Number of patients who received a CDK 4/6 inhibitor in the White and Black populations.

Characteristic	Black N(%)	White N(%)	Overall N(%)
**CDK 4/6i therapy**			
No	19 (26.03)	108 (18.27)	127 (19.13)
Yes	54 (73.97)	483 (81.73)	537 (80.87)
		**Fisher's exact**	0.12

Abbreviations: CDK 4/6i, CDK4/6 inhibitor

© 2025 Podany EL et al. *JAMA Network Open*.

eTable 12. Number of patients who received an mTOR inhibitor in the White and Black populations.

Characteristic	Black N(%)	White N(%)	Overall N(%)
**MTOR inhibitor use**			
No	55 (76.39)	447 (76.94)	502 (76.88)
Yes	17 (23.61)	134 (23.06)	151 (23.12)
		**Fisher's exact**	0.88

© 2025 Podany EL et al. *JAMA Network Open*.

eTable 13. Differences in A1c between Black and White patients with *PIK3CA* mutations.

	Black	White	P value
**Median A1c**	6.1	5.5	0.01

A1c value	Black N(%)	White N(%)	P value
**<= 6.4%**	12<br>(70.59%)	99<br>(84.62%)	0.15
**> 6.4%**	5<br>(29.41%)	18<br>(15.38%)	

© 2025 Podany EL et al. *JAMA Network Open*.

eTable 14. Univariate Cox regression analysis of clinical characteristics in the Black population.

Characteristic	HR	95% C.I.	P>|z|
**Lung**	2.19	1.32-3.63	0.002
**Liver**	2.82	1.71-4.65	<0.001
**Bone**	1.52	0.88-2.63	0.14
**Node**	1.02	0.62-1.70	0.92
**Soft tissue**	1.49	0.80-2.75	0.20
**CNS**	2.59	1.17-2.74	0.02
De novo	0.85	0.47-1.52	0.58
**ET type**			
Fulvestrant	0.45	0.24-0.83	0.01
AI	0.27	0.13-0.56	<0.001
**Treatment line**			
2	1.89	0.83-4.29	0.13
>=3	2.17	1.10-4.27	0.02

Abbreviations: OR, Odds Ratio; C.I., Confidence Interval; ET, Endocrine Therapy; CNS, central nervous system

© 2025 Podany EL et al. *JAMA Network Open*.

eTable 15. Univariate Cox regression analysis of single gene alterations in the Black population.

Alteration	HR	95% C.I.	P>|z|
**PIK3CA SNVs**	1.80	1.06-3.04	0.03
**ESR1 SNVs**	0.98	0.55-1.76	0.95
**TP53 SNVs**	1.56	0.94-3.59	0.08
**ARID1A SNVs**	0.67	0.16-2.74	0.58
**ERBB2 SNVs**	4.47	1.55-12.88	0.006
**CDKN2A SNVs**	6.47	1.93-21.65	0.002
**NF1 SNVs**	42.17	4.39-405.36	0.001
**PTEN SNVs**	3.39	1.20-9.59	0.02
**AKT1 SNVs**	3.04	0.93-9.89	0.06
**GATA3 SNVs**	1.13	0.55-2.29	0.74
**FBXW7 SNVs**	31.39	3.51-280.89	0.002
**HRAS SNVs**	9.20	1.20-70.36	0.03
**GNAS SNVs**	2.03	0.73-5.70	0.18
**BRCA2 SNVs**	2.25	0.54-9.47	0.27
**KRAS SNVs**	0.83	0.20-3.41	0.80
**NRAS SNVs**	2.32	0.32-16.93	0.41
**BRCA1 SNVs**	4.69	1.13-19.49	0.03
**BRAF SNVs**	1.24	0.17-8.98	0.83
**APC SNVs**	0.61	0.08-4.42	0.63
**JAK2 SNVs**	1.99	0.27-14.47	0.50
**STK11 SNVs**	1.84	0.25-13.41	0.54
**PTPN11 SNVs**	4.03	0.55-29.73	0.17
**NOTCH1 SNVs**	1.36	0.19-9.86	0.76
**KIT CNVs**	3.84	0.91-16.18	0.07
**PDGFRA CNVs**	7.49	2.22-25.24	0.001

© 2025 Podany EL et al. *JAMA Network Open*.

Alteration	HR	95% C.I.	P>|z|
**BRAF CNVs**	2.43	0.86-6.88	0.09
**ERBB2 CNVs**	0.94	0.29-3.01	0.91
**CCND1 CNVs**	1.95	0.99-3.84	0.05
**KRAS CNVs**	2.46	0.75-8.06	0.14
**AR CNVs**	3.98	0.95-16.64	0.06
**RAF1 CNVs**	1.04	0.25-4.27	0.96
**CDK6 CNVs**	2.92	0.70-12-10	0.14
**PIK3CA CNVs**	2.93	1.42-6.06	0.004
**EGFR CNVs**	2.68	1.26-5.68	0.01
**MYC CNVs**	3.45	1.70-6.97	0.001
**MET CNVs**	2.34	0.56-9.73	0.24
**CCNE1 CNVs**	1.33	0.42-4.26	0.63
**CCND2 CNVs**	4.30	1.80-10.30	0.001
**FGFR2 CNVs**	1.70	0.23-12.36	0.60

Abbreviations: SNVs, Single Nucleotide Variants; CNVs, Copy Number Variants; HR, Hazard Ratio; C.I., Confidence Interval

© 2025 Podany EL et al. *JAMA Network Open*.

eTable 16. Univariate Cox regression analysis of pathway alterations in the Black population.

Alteration	HR	95% C.I.	P>|z|
**PI3K SNVs**	2.33	1.40-3.89	0.001
**ER SNVs**	1.06	0.62-1.79	0.84
**P53 SNVs**	1.64	0.99-2.72	0.06
**RTK SNVs**	3.65	1.43-9.34	0.007
**Cell Cycle SNVs**	8.93	3.06-26.08	<0.001
**RAS SNVs**	1.88	0.75-4.73	0.18
**NOTCH SNVs**	2.63	0.64-10.87	0.18
**RAF SNVs**	0.93	0.13-6.74	0.94
**WNT SNVs**	0.61	0.08-4.43	0.63
**RTK CNVs**	1.75	1.01-3.04	0.05
**RAF CNVs**	1.71	0.73-3.99	0.22
**Cell Cycle CNVs**	2.34	1.35-4.05	0.003
**RAS CNVs**	2.46	0.75-8.06	0.14
**ER CNVs**	3.98	0.95-16.64	0.06
**PI3K CNVs**	2.93	1.42-6.06	0.004
**MYC CNVs**	3.45	1.70-6.98	0.001

Abbreviations: SNVs, Single Nucleotide Variants; CNVs, Copy Number Variants; HR, Hazard Ratio; C.I., Confidence Interval

© 2025 Podany EL et al. *JAMA Network Open*.

eTable 17. Univariate Cox regression analysis of clinical characteristics in the HR+/HER2- Black population.

Characteristic	HR	95% C.I.	P>|z|
**Lung**	1.75	0.94-3.25	0.08
**Liver**	4.14	2.19-7.84	<0.001
**Bone**	2.32	0.98-5.52	0.06
**Node**	0.90	0.48-1.68	0.74
**Soft tissue**	0.93	0.33-2.62	0.89
**CNS**	3.23	1.35-7.72	0.008
**De novo**	0.87	0.44-1.72	0.69
**Treatment line**			
2	1.47	10.57-3.84	0.43
>=3	1.56	0.69-3.48	0.28

Abbreviations: HR, Hazard Ratio; C.I., Confidence Interval; CNS, Central nervous system

© 2025 Podany EL et al. *JAMA Network Open*.

eTable 18. Univariate Cox regression analysis of single gene alterations in the HR+/HER2- Black population.

Alteration	HR	95% C.I.	P>|z|
**PIK3CA SNVs**	1.83	0.98-3.41	0.06
**ESR1 SNVs**	0.92	0.48-1.76	0.80
**TP53 SNVs**	1.73	0.91-3.27	0.09
**ARID1A SNVs**	0.34	0.05-2.50	0.29
**ERBB2 SNVs**	4.83	1.41-16.51	0.01
**CDKN2A SNVs**	4.05	0.93-17.51	0.06
**NF1 SNVs**	28.08	2.92-269.97	0.004
**PTEN SNVs**	3.64	1.09-12.17	0.04
**AKT1 SNVs**	4.14	1.24-13.84	0.02
**GATA3 SNVs**	0.99	0.46-2.16	0.99
**GNAS SNVs**	0.52	0.07-3.94	0.52
**KRAS SNVs**	0.92	0.22-3.81	0.90
**NRAS SNVs**	2.42	0.33-17.85	0.39
**APC SNVs**	1.82	0.25-13.40	0.56
**JAK2 SNVs**	2.02	0.27-14.86	0.49
**PTPN11 SNVs**	3.67	0.49-27.47	0.20
**NOTCH1 SNVs**	1.44	0.20-10.60	0.72
**BRAF SNVs**	1.34	0.18-9.84	0.77
**KIT CNVs**	7.79	1.76-34.53	0.007
**PDGFRA CNVs**	7.04	2.02-24.49	0.002
**BRAF CNVs**	2.07	0.28-15.43	0.48
**CCND1 CNVs**	2.89	1.40-5.96	0.004
**KRAS CNVs**	2.85	0.67-12.18	0.16
**AR CNVs**	5.10	0.67-38.64	0.11
**RAF1 CNVs**	0.64	0.09-4.75	0.66

© 2025 Podany EL et al. *JAMA Network Open*.

Alteration	HR	95% C.I.	P>|z|
**PIK3CA CNVs**	2.37	0.84-6.69	0.10
**EGFR CNVs**	2.96	1.23-7.13	0.01
**MYC CNVs**	4.26	1.80-10.10	0.001
**MET CNVs**	2.07	0.28-15.43	0.48
**CCND2 CNVs**	5.91	1.73-20.19	0.005

Abbreviations: SNVs, Single Nucleotide Variants; CNVs, Copy Number Variants; HR, Hazard Ratio; C.I., Confidence Interval

© 2025 Podany EL et al. *JAMA Network Open*.

eTable 19. Univariate Cox regression analysis of pathway alterations in the HR+/HER2- Black population.

Alteration	HR	95% C.I.	P>|z|
**PI3K SNVs**	2.38	1.27-4.44	0.007
**ER SNVs**	0.93	0.51-1.73	0.83
**P53 SNVs**	1.84	0.98-3.48	0.06
**RTK SNVs**	3.69	1.28-10.65	0.02
**Cell Cycle SNVs**	6.32	1.84-21.70	0.003
**RAS SNVs**	1.67	0.59-4.73	0.33
**NOTCH SNVs**	2.75	0.65-11.52	0.17
**RAF SNVs**	0.96	0.13-6.99	0.96
**WNT SNVs**	1.82	0.25-13.40	0.56
**RTK CNVs**	1.90	0.94-3.84	0.07
**RAF CNVs**	0.99	0.98-4.15	0.99
**Cell Cycle CNVs**	3.23	1.62-6.44	0.001
**RAS CNVs**	2.85	0.67-12.18	0.16
**ER CNVs**	5.10	0.67-38.64	0.11
**PI3K CNVs**	2.37	0.84-6.69	0.10
**MYC CNVs**	4.26	1.79-10.1’	0.001

Abbreviations: SNVs, Single Nucleotide Variants; CNVs, Copy Number Variants; HR, Hazard Ratio; C.I., Confidence Interval

© 2025 Podany EL et al. *JAMA Network Open*.

eTable 20. Multivariate Cox regression analysis of clinical characteristics and single gene alterations in the Black population.

Characteristic	HR	95% C.I.		P>|z|
**Lung**				
Yes	1.38	0.79	2.43	0.25
**Liver**				
Yes	2.21	1.25	3.92	0.007
**CNS**				
Yes	1.37	0.55	3.39	0.50
**Treatment Line**				
2	1.40	0.59	3.35	0.45
>=3	1.67	0.82	3.40	0.16
**PIK3CA SNVs**				
mut	1.23	0.68	2.22	0.40
**MYC CNVs**				
ampl	3.97	1.76	8.97	0.001

Abbreviations: SNVs, Single Nucleotide Variants; CNVs, Copy Number Variants; HR, Hazard Ratio; C.I., Confidence Interval; CNS, Central nervous system; mut, mutation; ampl, amplification

eTable 21. Multivariate Cox regression analysis of clinical characteristics and pathway alterations in the Black population.

Characteristic	HR	95% C.I.		P>|z|
**Lung**				
Yes	1.49	0.82	2.71	0.19
**Liver**				
Yes	2.18	1.21	3.93	0.009
**CNS**				
Yes	1.44	0.58	3.59	0.43
**Treatment line**				
2	1.57	0.62	4.00	0.34
>=3	1.88	0.90	3.96	0.09
**PI3K SNVs**	1.55	0.88	2.74	0.13
**RTK CNVs**	1.52	0.74	3.11	0.25
**Cell Cycle CNVs**	1.87	0.91	3.84	0.09
**MYC CNVs**	1.96	0.72	5.30	0.19

Abbreviations: SNVs, Single Nucleotide Variants; CNVs, Copy number variants; HR, Hazard Ratio; C.I., Confidence Interval; CNS, Central nervous system

© 2025 Podany EL et al. *JAMA Network Open*.

eTable 22. Multivariate Cox regression analysis of clinical characteristics and pathway alterations in the HR+/HER2- Black population.

**Characteristic**	**HR**	**95% conf. interval**		**P>|z|**
**Liver**				
Yes	3.90	2.01	7.54	<0.001
**CNS**				
Yes	2.07	0.84	5.07	0.11
**PI3K CNVS**	2.19	1.14	4.21	0.02
**Cell Cycle CNVs**	2.00	0.97	4.12	0.06

Abbreviations: CNVs, Copy number variants; HR, Hazard Ratio; C.I., Confidence Interval; CNS, Central nervous system

© 2025 Podany EL et al. *JAMA Network Open*.

eTable 23. Univariate Cox regression analysis of clinical characteristics in the White population.

	**HR**	**95% C.I.**	**P>|z|**
**Lung**	1.39	1.15-1.69	0.001
**Liver**	1.79	1.49-2.15	<0.001
**Bone**	1.36	1.11-1.68	0.003
**Node**	1.08	0.90-1.31	0.39
**Soft tissue**	1.50	1.20-1.89	<0.001
**CNS**	1.70	1.23-2.34	0.001
**De novo**	1.21	0.97-1.49	0.09
**ET type**			
Fulvestrant	0.53	0.43-0.67	<0.001
AI	0.36	0.28-0.47	<0.001
**Treatment line**			
2	2.01	1.50-2.70	<0.001
>=3	2.86	2.23-3.67	<0.001

Abbreviations: HR, Hazard Ratio; C.I., Confidence Interval; CNS, Central nervous system; ET, endocrine therapy; AI, aromatase inhibitor

© 2025 Podany EL et al. *JAMA Network Open*.

eTable 24. Univariate Cox regression analysis of single gene alterations in the White population.

Alteration	HR	95% C.I.	P>|z|
**PIK3CA SNVs**	1.26	1.04-1.53	0.020
**ESR1 SNVs**	1.89	1.54-2.32	<0.001
**TP53 SNVs**	1.96	1.63-2.35	<0.001
**ARID1A SNVs**	0.88	0.57-1.35	0.55
**ERBB2 SNVs**	1.29	0.82-2.02	0.26
**CDKN2A SNVs**	0.78	0.19-3.12	0.72
**NF1 SNVs**	1.73	2.08-2.77	0.02
**PTEN SNVs**	1.81	1.20-2.74	0.005
**CDH1 SNVs**	1.59	0.91-2.76	0.10
**AKT1 SNVs**	1.31	0.83-2.08	0.24
**GATA3 SNVs**	1.27	0.85-1.90	0.24
**SMAD4 SNVs**	1.70	1.05-2.75	0.03
**RB1 SNVs**	2.31	1.57-3.39	<0.001
**FBXW7 SNVs**	0.96	0.24-3.85	0.95
**HRAS SNVs**	5.23	2.59-10.56	<0.001
**GNAS SNVs**	1.40	0.75-2.61	0.30
**BRCA2 SNVs**	1.20	0.73-1.97	0.48
**IDH1 SNVS**	0.94	0.23-3.76	0.93
**KRAS SNVs**	0.81	0.44-1.47	0.48
**NRAS SNVs**	0.37	0.52-2.61	0.32
**BRCA1 SNVs**	0.79	0.38-1.68	0.55
**FGFR1 SNVs**	3.13	1.17-8.40	0.02
**BRAF SNVs**	1.65	0.91-2.99	0.10
**FGFR2 SNVs**	2.15	1.15-4.03	0.02
**RHOA SNVs**	1.85	0.76-4.47	0.17

© 2025 Podany EL et al. *JAMA Network Open*.

Alteration	HR	95% C.I.	P>|z|
**APC SNVs**	1.37	0.76-2.50	0.30
**JAK2 SNVs**	0.56	0.08-4.01	0.57
**RAF1 SNVs**	0.60	0.08-4.27	0.61
**STK11 SNVs**	1.35	0.56-3.25	0.51
**PTPN11 SNVs**	0.49	0.07-3.46	0.47
**ATM SNVs**	0.79	0.45-1.37	0.40
**KIT SNVs**	8.51	1.19-60.95	0.03
**RET SNVs**	0.89	0.22-3.59	0.87
**EGFR SNVs**	1.35	0.56-3.26	0.51
**TERT SNVs**	27.81	3.78-204.56	0.001
**IDH2 SNVs**	1.13	0.42-3.03	0.80
**MAPK1 SNVs**	1.31	0.18-9.33	0.79
**TCS1 SNVs**	1.22	0.30-4.90	0.78
**ARAF SNVs**	7.90	1.96-31.85	0.004
**PDGFRA SNVs**	0.44	0.06-3.13	0.41
**NFE2L2 SNVs**	0.85	0.21-3.42	0.82
**MPL SNVs**	1.12	0.16-8.01	0.91
**HNF1A SNVs**	0.94	0.13-6.72	0.95
**NOTCH1 SNVs**	6.26	0.88-44.78	0.07
**MAP2K2 SNVs**	47.48	6.39-352.98	<0.001
**KIT CNVs**	2.26	1.24-4.11	0.008
**PDGFRA CNVs**	1.86	1.07-3.24	0.03
**BRAF CNVs**	2.86	2.00-4.09	<0.001
**ERBB2 CNVs**	0.89	0.55-1.44	0.63
**CDK4 CNVs**	1.56	0.86-2.84	0.14
**CCND1 CNVs**	2.27	1.69-3.05	<0.001

© 2025 Podany EL et al. *JAMA Network Open*.

Alteration	HR	95% C.I.	P>|z|
**KRAS CNVs**	2.35	1.53-3.61	<0.001
**AR CNVs**	2.01	1.07-3.76	0.03
**RAF1 CNVs**	2.18	1.38-3.45	0.001
**CDK6 CNVs**	1.84	1.21-2.81	0.004
**PIK3CA CNVs**	2.57	1.94-3.40	<0.001
**EGFR CNVs**	2.17	1.63-2.89	<0.001
**MYC CNVs**	2.01	1.22-3.31	0.006
**MET CNVs**	1.18	0.26-5.27	0.83
**CCNE1 CNVs**	2.03	1.42-2.89	<0.001
**CCND2 CNVs**	2.30	1.23-4.32	0.009
**FGFR2 CNVs**	3.99	2.24-7.11	<0.001

Abbreviations: SNVs, Single Nucleotide Variants; CNVs, Copy number variants; HR, Hazard Ratio; C.I., Confidence Interval

© 2025 Podany EL et al. *JAMA Network Open*.

eTable 25. Univariate Cox regression analysis of pathways alterations in the White population.

Alteration	HR	95% C.I.	P>|z|
**PI3K SNVs**	1.33	1.10-1.60	0.003
**ER SNVs**	1.70	1.39-2.08	<0.001
**P53 SNVs**	1.86	1.55-2.24	<0.001
**RTK SNVs**	1.43	1.04-1.96	0.03
**Cell Cycle SNVs**	1.96	1.37-2.88	<0.001
**RAS SNVs**	1.31	0.93-1.83	0.12
**NOTCH SNVs**	1.34	0.43-4.17	0.61
**RAF SNVs**	1.63	0.96-2.79	0.07
**WNT SNVs**	1.34	0.74-2.44	0.34
**MEK SNVs**	2.45	0.78-7.63	0.12
**NRF2 SNVs**	0.85	0.21-3.42	0.82
**RTK CNVs**	1.73	1.40-2.13	<0.001
**RAF CNVs**	2.73	2.01-3.70	<0.001
**Cell Cycle CNVs**	2.13	1.70-2.67	<0.001
**RAS CNVs**	2.35	1.53-3.61	<0.001
**ER CNVs**	2.05	1.12-3.73	0.02
**PI3K CNVs**	2.57	1.94-3.40	<0.001
**MYC CNVs**	2.50	1.91-3.30	<0.001

Abbreviations: SNVs, Single Nucleotide Variants; CNVs, Copy number variants; HR, Hazard Ratio; C.I., Confidence Interval

© 2025 Podany EL et al. *JAMA Network Open*.

eTable 26. Univariate Cox regression analysis of clinical characteristics in the HR+/HER2- White population.

Characteristic	HR	95% C.I.	P>|z|
**Lung**	1.28	1.01-1.62	0.04
**Liver**	2.14	1.73-2.66	<0.001
**Bone**	1.64	1.23-2.18	0.001
**Node**	0.98	0.78-1.23	0.87
**Soft tissue**	1.60	1.20-2.12	0.001
**CNS**	2.03	1.32-3.13	0.001
**De novo**	1.39	1.08-1.79	0.01
**Treatment line**			
2	2.22	1.53-3.21	<0.001
>=3	3.86	2.81-5.30	<0.001

Abbreviations: HR, Hazard Ratio; C.I., Confidence Interval; CNS, Central nervous system; ET, endocrine therapy; AI, aromatase inhibitor

© 2025 Podany EL et al. *JAMA Network Open*.

eTable 27. Univariate Cox regression analysis of single gene alterations in the HR+/HER2- White population.

Alteration	HR	95% C.I.	P>|z|
**PIK3CA SNVs**	1.24	0.99-1.55	0.06
**ESR1 SNVs**	2.06	1.64-2.59	<0.001
**TP53 SNVs**	1.77	1.43-2.20	<0.001
**ARID1A SNVs**	0.88	0.55-1.40	0.58
**ERBB2 SNVs**	1.83	1.09-3.08	0.02
**CDKN2A SNVs**	0.84	0.21-3.38	0.81
**NF1 SNVs**	2.51	1.49-4.22	0.001
**PTEN SNVs**	1.96	1.18-3.25	0.009
**CDH1 SNVs**	1.60	0.89-2.86	0.11
**AKT1 SNVs**	1.36	0.82-2.24	0.24
**GATA3 SNVs**	1.31	0.85-1.99	0.22
**SMAD4 SNVs**	2.07	1.21-3.54	0.008
**RB1 SNVs**	2.02	1.29-3.18	0.002
**FBXW7 SNVs**	0.86	0.12-6.13	0.88
**HRAS SNVs**	4.99	2.35-10.61	<0.001
**GNAS SNVs**	1.27	0.60-2.69	0.53
**BRCA2 SNVs**	1.16	0.65-2.07	0.60
**IDH1 SNVS**	0.61	0.09-4.35	0.62
**KRAS SNVs**	0.70	0.35-1.41	0.31
**BRCA1 SNVs**	0.71	0.27-1.91	0.50
**FGFR1 SNVs**	3.24	1.20-8.71	0.02
**BRAF SNVs**	1.83	0.97-3.44	0.06
**FGFR2 SNVs**	2.04	1.01-4.11	0.05
**RHOA SNVs**	1.54	0.57-4.14	0.39
**APC SNVs**	1.39	0.69-2.81	0.36

© 2025 Podany EL et al. *JAMA Network Open*.

Alteration	HR	95% C.I.	P>|z|
**JAK2 SNVs**	0.56	0.08-3.97	0.56
**RAF1 SNVs**	0.64	0.09-4.55	0.65
**STK11 SNVs**	1.08	0.35-3.36	0.90
**PTPN11 SNVs**	1.11	0.16-7.92	0.92
**ATM SNVs**	0.71	0.35-1.43	0.33
**KIT SNVs**	9.28	1.29-66.81	0.03
**RET SNVs**	0.90	0.23-3.64	0.89
**EGFR SNVs**	0.85	0.21-3.41	0.82
**TERT SNVs**	30.06	4.00-225.62	0.001
**IDH2 SNVs**	0.61	0.15-2.47	0.492
**MAPK1 SNVs**	1.36	0.19-9.72	0.76
**TCS1 SNVs**	1.24	0.31-5.01	0.75
**ARAF SNVs**	8.31	2.05-33.69	0.003
**PDGFRA SNVs**	0.42	0.06-3.02	0.39
**NFE2L2 SNVs**	0.84	0.21-3.37	0.80
**MPL SNVs**	1.11	0.16-7.92	0.92
**HNF1A SNVs**	0.94	0.13-6.72	0.95
**MAP2K2 SNVs**	42.67	5.66-321.73	<0.001
**BRAF SNVs**	1.83	0.97-3.44	0.06
**KIT CNVs**	2.77	1.03-7.47	0.04
**PDGFRA CNVs**	2.21	1.04-4.68	0.04
**BRAF CNVs**	2.04	1.17-3.56	0.01
**ERBB2 CNVs**	2.55	1.05-6.17	0.04
**CDK4 CNVs**	1.30	0.58-2.92	0.52
**CCND1 CNVs**	2.13	1.51-2.99	<0.001
**KRAS CNVs**	1.82	0.99-3.32	0.05

© 2025 Podany EL et al. *JAMA Network Open*.

Alteration	HR	95% C.I.	P>|z|
**AR CNVs**	2.63	1.17-5.90	0.02
**RAF1 CNVs**	2.61	1.53-4.47	0.001
**CDK6 CNVs**	1.22	0.65-2.28	0.54
**PIK3CA CNVs**	2.17	1.51-3.10	<0.001
**EGFR CNVs**	1.80	1.24-2.61	0.002
**MYC CNVs**	2.71	1.93-3.81	<0.001
**MET CNVs**	1.18	0.52-2.64	0.69
**CCNE1 CNVs**	2.14	1.27-3.59	0.004
**CCND2 CNVs**	1.67	0.62-4.48	0.31
**FGFR2 CNVs**	4.07	2.01-8.28	<0.001

Abbreviations: SNVs, Single Nucleotide Variants; CNVs, Copy number variants; HR, Hazard Ratio; C.I., Confidence Interval

© 2025 Podany EL et al. *JAMA Network Open*.

eTable 28. Univariate Cox regression analysis of pathway alterations in the HR+/HER2- White population.

Alteration	HR	95% C.I.	P>|z|
**PI3K SNVs**	1.33	1.07-1.65	0.01
**ER SNVs**	1.87	1.50-2.34	<0.001
**P53 SNVs**	1.65	1.33-2.05	<0.001
**RTK SNVs**	1.60	1.11-2.30	0.01
**Cell Cycle SNVs**	1.74	1.13-2.68	0.01
**RAS SNVs**	1.61	1.10-2.34	0.01
**NOTCH SNVs**	0.76	0.11-5.44	0.79
**RAF SNVs**	1.80	1.03-3.13	0.04
**WNT SNVs**	1.38	0.67-3.72	0.40
**MEK SNVs**	2.64	0.66-10.69	0.17
**NRF2 SNVs**	0.84	0.21-3.37	0.80
**RTK CNVs**	1.68	1.29-2.19	<0.001
**RAF CNVs**	2.35	1.55-3.57	<0.001
**Cell Cycle CNVs**	1.86	1.40-2.48	<0.001
**RAS CNVs**	1.82	0.99-3.32	0.05
**ER CNVs**	2.43	1.08-5.46	0.03
**PI3K CNVs**	2.17	1.51-3.10	<0.001
**MYC CNVs**	2.71	1.93-3.81	<0.001

Abbreviations: SNVs, Single Nucleotide Variants; CNVs, Copy number variants; HR, Hazard Ratio; C.I., Confidence Interval

© 2025 Podany EL et al. *JAMA Network Open*.

eTable 29. Multivariate Cox regression analysis of clinical characteristics and single gene alterations in the White population.

Characteristic	HR	95% C.I.		P>|z|
**Lung**				
Yes	1.17	0.95	1.45	0.13
**Liver**				
Yes	1.49	1.21	1.83	<0.001
**Bone**				
Yes	1.29	1.01	1.65	0.04
**Soft tissue**				
Yes	1.52	1.18	1.96	0.001
**CNS**				
Yes	1.47	1.03	2.11	0.03
**Treatment line**				
2	1.80	1.32	2.46	<0.001
3	2.39	1.84	3.11	<0.001
**PIK3CA SNVs**				
mut	0.96	0.77	1.20	0.75
**ESR1 SNVs**				
mut	1.39	1.10	1.76	0.007
**TP53 SNVs**				
mut	1.79	1.46	2.19	<0.001
**NF1 SNVs**				
mut	1.17	0.68	2.00	0.58
**PTEN SNVs**				
mut	1.24	0.77	2.02	0.38

© 2025 Podany EL et al. *JAMA Network Open*.

Characteristic	HR	95% C.I.		P>|z|
**SMAD4 SNVs**				
mut	1.34	0.76	2.37	0.31
**RB1 SNVs**				
mut	1.04	0.65	1.65	0.88
**KIT CNVs**				
ampl	1.46	0.43	4.92	0.54
**AR CNVs**				
ampl	0.72	0.32	1.63	0.43
**PDGFRA CNVs**				
ampl	0.61	0.20	1.87	0.39
**BRAF CNVs**				
ampl	2.39	1.37	4.18	0.002
**CCND1 CNVs**				
ampl	1.43	0.99	2.06	0.06
**KRAS CNVs**				
ampl	0.99	0.57	1.72	0.99
**RAF1 CNVs**				
ampl	1.37	0.68	2.79	0.38
**CDK6 CNVs**				
ampl	1.36	0.81	2.29	0.24
**PIK3CA CNVs**				
ampl	1.35	0.93	1.96	0.11
**EGFR CNVs**				
ampl	1.21	0.81	1.81	0.35
**MYC CNVs**				
ampl	1.49	1.03	2.16	0.03

© 2025 Podany EL et al. *JAMA Network Open*.

Characteristic	HR	95% C.I.		P>|z|
**MET CNVs**				
ampl	0.52	0.23	1.18	0.12
**CCNE1 CNVs**				
ampl	0.97	0.60	1.58	0.90
**CCND2 CNVs**				
ampl	0.96	0.39	2.38	0.93

Abbreviations: SNVs, Single Nucleotide Variants; CNVs, Copy number variants; HR, Hazard Ratio; C.I., Confidence Interval; CNS, Central nervous system; mut, mutation; ampl, amplification

© 2025 Podany EL et al. *JAMA Network Open*.

eTable 30. Multivariate Cox regression analysis of clinical characteristics and single gene alterations in the HR+/HER2- White population.

Characteristic	HR	95% C.I.		P>|z|
**Lung**				
Yes	1.01	0.78	1.31	0.93
**Liver**				
Yes	1.79	1.40	2.28	<0.001
**Bone**				
Yes	1.35	0.97	1.87	0.07
**Soft tissue**				
Yes	1.63	1.19	2.23	0.002
**CNS**				
Yes	2.14	1.33	3.45	0.002
**De Novo**	1.25	0.95	1.65	0.11
**Treatment line**				
2	1.68	1.13	2.49	0.01
>=3	2.67	1.91	3.75	<0.001
**ESR1 SNVs**				
mut	1.42	1.09	1.85	0.01
**ERBB2 SNVs**				
mut	1.31	0.69	2.50	0.40
**TP53 SNVs**				
mut	1.53	1.20	1.94	0.001
**NF1 SNVs**				
mut	1.90	1.06	3.41	0.03
**PTEN SNVs**				
mut	1.45	0.81	2.62	0.21

© 2025 Podany EL et al. *JAMA Network Open*.

Characteristic	HR	95% C.I.	P>|z|	
**SMAD4 SNVs**				
mut	1.43	0.74	2.75	0.28
**RB1 SNVs**				
mut	0.87	0.50	1.51	0.62
**BRAF CNVs**				
ampl	1.22	0.56	2.64	0.61
**CCND1 CNVs**				
ampl	1.54	1.03	2.30	0.04
**RAF1 SNVs**				
ampl	1.21	0.55	2.67	0.64
**PIK3CA CNVs**				
ampl	1.4	0.88	2.23	0.15
**EGFR CNVs**				
ampl	0.92	0.56	1.52	0.75
**MYC CNVs**				
ampl	2.04	1.30	3.20	0.002
**CCNE1 CNVs**				
ampl	0.78	0.40	1.52	0.46

Abbreviations: SNVs, Single Nucleotide Variants; CNVs, Copy number variants; HR, Hazard Ratio; C.I., Confidence Interval; CNS, Central nervous system; mut, mutation; ampl, amplification

© 2025 Podany EL et al. *JAMA Network Open*.

eTable 31. Multivariate Cox regression analysis of clinical characteristics and pathway alterations in the White population.

**Characteristic**	**HR**	**95% C.I.**		**P>|z|**
**Lung**				
Yes	1.15	0.94	1.42	0.17
**Liver**				
Yes	1.48	1.21	1.81	<0.001
**Bone**				
Yes	1.28	1.01	1.64	0.04
**Soft tissue**				
Yes	1.49	1.16	1.90	0.002
**CNS**				
Yes	1.42	1.00	2.03	0.05
**Treatment line**				
2	1.90	1.40	2.59	<0.001
3	2.49	1.92	3.22	<0.001
**PI3K SNVs**	1.06	0.86	1.31	0.58
**ER SNVs**	1.22	0.97	1.54	0.09
**P53 SNs**	1.73	1.41	2.12	<0.001
**Cell Cycle SNVs**	1.07	0.70	1.64	0.76
**RTK SNVs**	1.30	0.92	1.86	0.14
**RAS SBVs**	1.02	0.70	1.48	0.93
**RTK CNVs**	0.99	0.74	1.31	0.93
**RAF CNVs**	1.65	1.08	2.54	0.02
**Cell Cycle CNVs**	1.30	0.97	1.73	0.07
**RAS CNVs**	0.91	0.55	1.52	0.72
**ER CNVs**	0.88	0.43	1.82	0.74
**PI3K CNVs**	1.52	1.07	2.15	0.02
**MYC CNVs**	1.36	0.94	1.97	0.10

Abbreviations: SNVs, Single Nucleotide Variants; CNVs, Copy number variants; HR, Hazard Ratio; C.I., Confidence Interval; CNS, Central nervous system

© 2025 Podany EL et al. *JAMA Network Open*.

eTable 32. Multivariate Cox regression analysis of clinical characteristics and pathway alterations in the HR+/HER2- White population.

Characteristic	HR	95% C.I.		P>|z|
**Lung**				
Yes	0.97	0.75	1.27	0.85
**Liver**				
Yes	1.82	1.42	2.31	<0.001
**Bone**				
Yes	1.43	1.03	2.00	0.03
**Soft tissue**				
Yes	1.55	1.14	2.12	0.005
**CNS**				
Yes	2.011	1.24	3.22	0.004
**De Novo**	1.17	0.90	1.54	0.24
**Treatment line**				
2	1.85	1.25	2.73	0.002
>=3	3.00	2.15	4.19	<0.001
**PIK3K SNVs**	1.07	0.83	1.36	0.61
**ER SNVs**	1.22	0.94	1.57	0.14
**P53 SNVs**	1.47	1.16	1.86	0.001
**RTK SNVs**	0.97	0.69	1.35	0.84
**Cell Cycle SNVs**	1.00	0.60	1.65	0.99
**RAS SNVs**	1.25	0.82	1.90	0.30
**RAF SNVs**	0.96	0.50	1.85	0.90
**RAF CNVs**	1.08	0.59	1.99	0.79
**Cell Cycle CNVs**	1.15	0.81	1.63	0.43
**PI3K CNVs**	1.60	1.04	2.45	0.03
**MYC CNVs**	1.93	1.24	2.99	0.003

Abbreviations: SNVs, Single Nucleotide Variants; CNVs, Copy number variants; HR, Hazard Ratio; C.I., Confidence Interval; CNS, Central nervous system

© 2025 Podany EL et al. *JAMA Network Open*.
