# Supplementary material for: Racial Differences in ctDNA Profiles, Targeted Therapy Use, and Outcomes in Metastatic Breast Cancer
Source: JAMA Netw Open. 2025 Feb 26;8(2):e2461899. doi: 10.1001/jamanetworkopen.2024.61899 (PMC11866032; doi:10.1001/jamanetworkopen.2024.61899)
Supplement: Supplement 2. — Data Sharing Statement [file jamanetwopen-e2461899-s002.pdf]

Data Sharing Statement

Podany. Racial Differences in ctDNA Profiles, Targeted Therapy Use, and Outcomes in Metastatic Breast Cancer. *JAMA Netw Open*. Published February 26, 2025. doi:10.1001/jamanetworkopen.2024.61899

Data
Data available: Yes
Data types: Deidentified participant data
How to access data: It will be available by request from Lorenzo Gerratana (lorenzo.gerratana@uniud.it) and Andrew A. Davis (aadavis@wustl.edu).
When available: With publication

Supporting Documents
Document types: None

Additional Information
Who can access the data: Any researchers requesting the cohort data. The validation dataset would need permission by researchers from the senior author and Guardant Health.
Types of analyses: For any purpose.
Mechanisms of data availability: A deidentified data spreadsheet will be uploaded in excel or csv format as a supplement.
